# Enhancer–promoter interactions become more instructive in the transition from cell-fate specification to tissue differentiation

**DOI:** 10.1038/s41588-024-01678-x

**Published:** 2024-03-11

**Authors:** Tim Pollex, Adam Rabinowitz, Maria Cristina Gambetta, Raquel Marco-Ferreres, Rebecca R. Viales, Aleksander Jankowski, Christoph Schaub, Eileen E. M. Furlong

**Affiliations:** 1 European Molecular Biology Laboratory (EMBL), Genome Biology Unit, Heidelberg, Germany; 2 Present Address: European Molecular Biology Laboratory (EMBL), Directors’ Research Unit, Heidelberg, Germany; 3 Present Address: Center for Integrative Genomics, University of Lausanne, Lausanne, Switzerland; 4 Present Address: Faculty of Mathematics, Informatics and Mechanics, University of Warsaw, Warsaw, Poland

**Keywords:** Gene regulation, Cell lineage

## Abstract

To regulate expression, enhancers must come in proximity to their target gene. However, the relationship between the timing of enhancer–promoter (E–P) proximity and activity remains unclear, with examples of uncoupled, anticorrelated and correlated interactions. To assess this, we selected 600 characterized enhancers or promoters with tissue-specific activity in *Drosophila* embryos and performed Capture-C in FACS-purified myogenic or neurogenic cells during specification and tissue differentiation. This enabled direct comparison between E–P proximity and activity transitioning from OFF-to-ON and ON-to-OFF states across developmental conditions. This showed remarkably similar E–P topologies between specified muscle and neuronal cells, which are uncoupled from activity. During tissue differentiation, many new distal interactions emerge where changes in E–P proximity reflect changes in activity. The mode of E–P regulation therefore appears to change as embryogenesis proceeds, from largely permissive topologies during cell-fate specification to more instructive regulation during terminal tissue differentiation, when E–P proximity is coupled to activation.

## Abstract (web summary)

Analysis of enhancer–promoter (E–P) interactions during *Drosophila* embryogenesis suggests that the relationship between E–P proximity and activity depends on the developmental stage. Increased E–P proximity is associated with activity during differentiation but not specification.

## Main

How enhancers convey regulatory information to their target genes has been intensely studied. The prevailing model involves spatial proximity between the enhancer and promoter (E–P); however, the distance required and its relationship to activity remain unclear. In some cases, the enhancer only comes into proximity (or interacts) with the gene’s promoter in the appropriate cell type and developmental stage where the gene is expressed^1–7^, termed an instructive loop^8^. For example, comparing mouse embryonic stem cells to in vitro differentiated, or in vivo isolated, cortical neurons, many putative enhancers (H3K27ac-positive regions) interact with promoters specifically at the stage when the gene was expressed^6^. Cell type- and stage-specific chromatin interactions have also been observed during cardiac development^5^, adipocyte differentiation^9^ and at rhythmically expressed loci^10^. In such an instructive mode of regulation, E–P proximity is highly correlated with enhancer activity and gene expression.

However, there is also evidence that E–P interactions can function in a more permissive manner, where their proximity is temporally and/or spatially (tissue) separated from transcriptional activation. Comparing the proximity of embryonic enhancers that are active during mesoderm specification to an earlier postgastrulation stage of *Drosophila* embryogenesis showed that the vast majority of tested enhancers were already in proximity to their target promoter hours before gene activation, despite changes in enhancer activity^11^. Such a permissive mode^8^, characterized by preformed E–P loops in the absence of gene expression, has also been observed during zebrafish^12^, mammalian macrophage^13^ and limb^14^ development, for example, the *HoxD* cluster^15,16^, and cell culture models during *trans*-differentiation^17^ and induced pluripotent stem cells reprogramming^18^, and is suggested to poise the system for rapid activation^11^. In line with this, preformed E–P loops have been observed in the context of inducible gene expression^19,20^. For example, the vast majority of genes activated upon neuronal stimulation had preformed E–P interactions before stimulation^21^. In some cases, both permissive and instructive modes of regulation occur^13,14,22^.

A third mode of regulation posits that E–P proximity is not required for activation^23^ or even anticorrelated with activity, as the E–P move further apart during activation^24,25^. How permissive, instructive or anticorrelated/noncorrelated loops are regulated remains unclear and may involve ubiquitously expressed transcription factors (TFs) for permissive interactions, as suggested for CTCF (CCCTC-binding factor)/cohesin^14^, while lineage- or stimulus-specific TFs are associated with both instructive^22,26,27^ and permissive^22^ interactions. It is also not clear why one mode of E–P communication is used in one context and not another—there are no obvious links to a particular gene function or tissue type and perhaps it reflects differences in the approaches taken.

Many studies started with a chromatin conformation capture experiment and then used chromatin signatures (for example, H3K27ac and p300)^6,9,12,13^ or promoter proximity itself (from Capture-C)^5,10,14^ to define putative enhancers, which may bias findings to enhancers in an active state or already in proximity. To more directly assess the relationship between E–P proximity and activity an orthogonal approach is needed, starting from enhancers (and promoters) with characterized activity in vivo and then measuring their proximity as they transition from an OFF-to-ON or ON-to-OFF state. To address this, we hand-selected ~600 regulatory elements (~300 embryonic enhancers and ~300 promoters) with characterized tissue-specific activity in vivo in either the embryonic muscle or nervous system. E–P proximity (interactions) were measured using Capture-C in purified muscle or neuronal nuclei during cell-fate specification and tissue differentiation in *Drosophila* embryos when these regulatory elements are in an ON or OFF state. This high-resolution view of hundreds of enhancers uncovered surprisingly similar E/P topologies between myogenic and neuronal lineages during cell-fate specification regardless of the activity state, with the permissive mode prevailing. At later stages, during terminal tissue differentiation, E/P usage switches to a more instructive mode, where many new, more distal E–P loops emerge. Here E–P proximity is associated with a gain in activity and vice versa, suggesting functional regulatory events, which we confirmed in transgenic embryos. These differences could not be explained by insulator binding. The alternative usage of predominantly permissive E–P topologies to more instructive regulation at later stages may enable plasticity during cell-fate decisions while ensuring diversification during terminal tissue differentiation.

## Results

### Quantifying E–P interactions in different tissues and stages

**Fig. 1:**
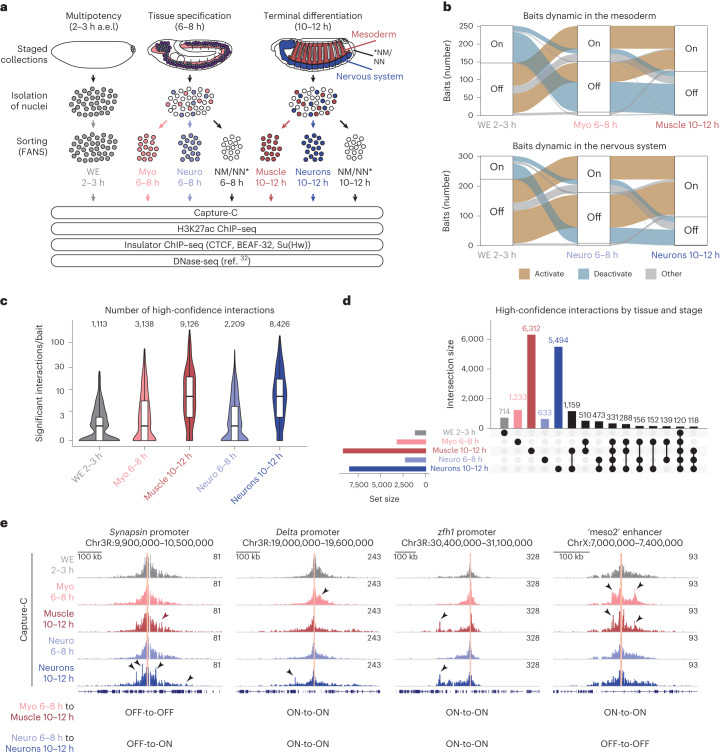
Quantifying E–P interactions across tissues and developmental time. **a**, Experimental overview, myogenic (Mef2^+^) and neuronal (Elav^+^) cells were isolated (>95% purity) from tightly staged embryos at 6-8 h and 10-12 h and used for Capture-C and ChIP–seq (H3K27ac, CTCF, BEAF-32, Su(Hw)) in biological replicates. Gray = WE 2–3 h, red = myogenic mesoderm (myo 6–8 h, muscle 10–12 h), blue = nervous system (neuro 6–8 h, neurons 10–12 h). NM/NN (Mef2^−^ and Elav^−^ cells). **b**, Alluvial plots showing the dynamic activity of the E–P baits in muscle and/or neuronal tissues (or both tissues). The number of E–Ps with activation (OFF–ON, brown) or deactivation (ON–OFF, blue) between conditions is indicated by the thickness of the lines. **c**, Violin plot/boxplot of the number of high-confidence interactions (CHiCAGO score ≥5 and DHS overlap) per bait at the indicated developmental time/tissue (colors as in **a**). Total number of high-confidence interactions for all baits indicated above. Boxplot: center = median; upper and lower bounds = interquartile range; whiskers = minimum and maximum. **d**, UpSet plot showing high-confidence E–P interactions in the five conditions. Unique interactions for each condition are indicated by colored bars (as in **a**). The 15 most frequent combinations are shown. **e**, Normalized Capture-C counts at three selected developmentally regulated genes (promoter baits) and one selected enhancer bait, highlighted in light pink. Gene names and genomic regions are indicated above; tissue and stage are indicated on the left (colors as in **a**) and the activity of each element in each condition (ON-to-OFF, etc.) is also indicated. Arrowheads indicate regions of interest with significantly differential interaction counts between tissues/stages.

The developmental enhancers or promoters (*n* = 600) were hand-selected from in vivo validated enhancers in transgenic embryos^28–30^ and genes with characterized expression by in situ hybridization^31^ (Fig. 1a,b and Supplementary Fig. 1a,b). The E–Ps were selected based on their dynamic tissue-specific activity, going from OFF-to-ON or ON-to-OFF in the myogenic (myoblast (myo) or differentiated muscle) or neurogenic (neuro or differentiated neurons) lineages (Fig. 1b and Supplementary Fig. 1a,b). Capture-C was performed on nuclei isolated from five developmental contexts (Fig. 1a): (i) early blastoderm whole embryos (WEs; 2–3 h, mainly early stage 5), mid-stage embryos during specification of myogenic (ii) and neuronal (iii) lineages (6–8 h, ~stage 10/11) and later-stage embryos during the initiation of terminal tissue differentiation of muscle (iv) and neurons (v) (10–12 h, ~stage 13). Isolated nuclei from the latter two time points were stained with antibodies for a nuclear marker specific for myoblast/muscle cells (Mef2) or developing and differentiating neurons (Elav), and fluorescence-activated nuclear sorted to >95% purity^32^ and used for Capture-C (Fig. 1a; Methods).

The baits (the 600 E/P regions targeted by the chromatin capture) were divided into libraries targeting enhancers or promoters separately, with 26 baits in common to determine reproducibility. Capture-C (using a 4 bp cutter) was performed on two replicates per tissue and time point, resulting in 20 datasets (Fig. 1a). The capture efficiency was largely comparable across conditions, and between the 26 common baits, attesting to the data reproducibility (Supplementary Figs. 1c–h and 2; Methods). The 583 baits that passed quality control (QC) represent 303 enhancers, 276 promoters and 4 regions overlapping both (Supplementary Table 1).

To obtain an overview of E–P interactions throughout all conditions, we defined a high-confidence set based on (1) an observed interaction frequency greater than background (modeled by CHiCAGO (Capture Hi-C Analysis of Genomic Organization)^33^, using a stringent score of ≥5), (2) overlap with a DHS (DNase-hypersensitive site) from the same time point/tissue^32^ to remove bystander interacting fragments and (3) removal of interacting regions very proximal (<2 kb) or distal (>10 Mb) to the bait (Methods). This identified 24,012 high-confidence interactions across all baits in one or more conditions, representing 18,252 unique interactions (Fig. 1c and Supplementary Data 1).

**Extended Data Fig. 1:**
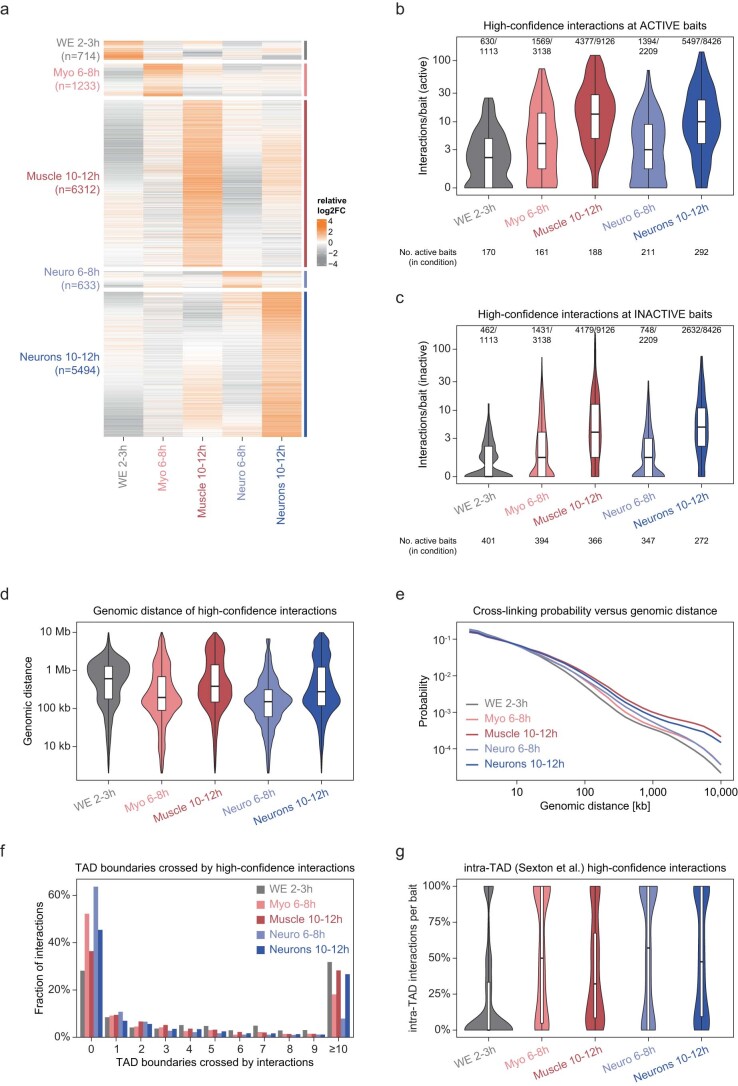
Properties of high-confidence E/P interactions. **(a)** Clustering of interaction frequencies at all high-confidence E/P interactions only called in one condition (colored bars in Fig. 1d), showing increase (orange) or decrease (gray) relative to the sample average. The majority of unique interactions have the highest interaction frequency in the condition (tissue/time point) where they were called significant (Fig. 1d) compared to all others. **(b**,**c)** Violin plots/boxplots of the number of high-confidence interactions (CHiCAGO score ≥5 and DHS overlap) per bait at the indicated developmental time/tissue for E/P baits active (**b**) or inactive (**c**) in the indicated condition. Number of interactions for active (**b**) or inactive (**c**) baits, as well as the total number of high-confidence interactions for all baits indicated above. Number of baits active (**b**) or inactive (**c**) is indicated below the plot. **(d)** Violin plots/boxplots displaying the distribution of genomic distances between the bait and ‘other end’ for all high-confidence interactions (CHiCAGO score ≥5, DHS overlap) identified in the 5 conditions. **(e)** P(s) plot displaying the probability of observing interactions at a given distance/separation between the bait and ‘other end’. Over developmental time there are fewer proximal interactions (<10 kb) and more distal interactions (>10 kb). In the identification of differential interactions (DESeq2 analysis; Methods), a normalization process was applied to account for these differences in the P(s) curves. **(f)** Bar chart depicting the fraction of high-confidence interactions per bait (y-axis) crossing TAD boundaries (x-axis, up to ≥10 boundaries) based on boundary annotation from whole embryos from ref. ^36^. **(g)** Violin plots/boxplots displaying the fraction of intra-TAD high-confidence interactions per baits in the 5 conditions. TAD annotation was based on ref. ^36^. For boxplots in **b**, **c**, **d**, **g**: center = median, upper and lower bounds = interquartile range, whiskers = minimum and maximum).

The number of high-confidence E/P interactions increases as development proceeds, ranging from ~1,000 to 3,000 going from 2–3 h (WE) to 6–8 h (in both myo or neuro; Fig. 1c) and from ~3,000 to 9,000 between 6–8 h and 10–12 h, moving from specification to tissue differentiation (Fig. 1c,d). This trend for the emergence of more interactions at 10–12 h is also clear from the quantitative signal in one condition (color bars; Fig. 1d) across all other conditions (Extended Data Fig. 1a). Moreover, the complexity of interactions, as seen by the number of interactions per bait, also increases (Fig. 1c)—median of 1 per bait during specification (6–8 h) compared to 7 during differentiation (10–12 h), in both tissues (Fig. 1c), with some E/Ps having over 30 high-confidence interactions.

Active E/P baits have more high-confidence interactions than inactive baits in the same condition (Extended Data Fig. 1b,c). Within the active baits, this increases dramatically between the stages of specification (6–8 h) to differentiation (10–12 h; Extended Data Fig. 1b). There is also an increase in the distance of interactions between specification and differentiation, with more distal interactions emerging at the later time point (10–12 h; Extended Data Fig. 1d,e). The 2–3 h time point behaves differently (Supplementary Note). The majority of E–P interactions are contained within a TAD (topologically associating domain) in all conditions, as expected, while a fraction cross at least one TAD border (Extended Data Fig. 1f,g), similar to the *Drosophila* *twist*^34^ and mouse *Sox2* (ref. ^35^) loci. Some E–P interactions cross over ten boundaries and represent long-range loops over megabase scales^36–39^.

This trend for increased E/P interactions during tissue differentiation is exemplified in five loci (Fig. 1e). *Synapsin* is expressed in differentiated neurons^40^. The *Synapsin* promoter has several significant interactions specifically in neuronal cells in the later differentiation time point (Fig. 1e (left, arrowheads)). *Delta* has very dynamic expression in both the myogenic and neuronal lineages^31^, which is reflected in the tissue- and stage-specific promoter interactions (Fig. 1e (middle, arrowheads)). *Zfh1 (Zn finger homeodomain 1*) is expressed in both tissues and time points, yet there are only significant interaction changes at the differentiation stage (Fig. 1e (right, arrowheads)). The meso2 enhancer is active in the early (6–8 h) and late stage (10–12 h) myogenic mesoderm^28^ and interacts with the promoter of *Chronophage*, a gene expressed in the somatic muscle and other tissues, suggesting that meso2 is a *Chronophage* enhancer.

### E–P proximity is coupled to activity during differentiation

**Fig. 2:**
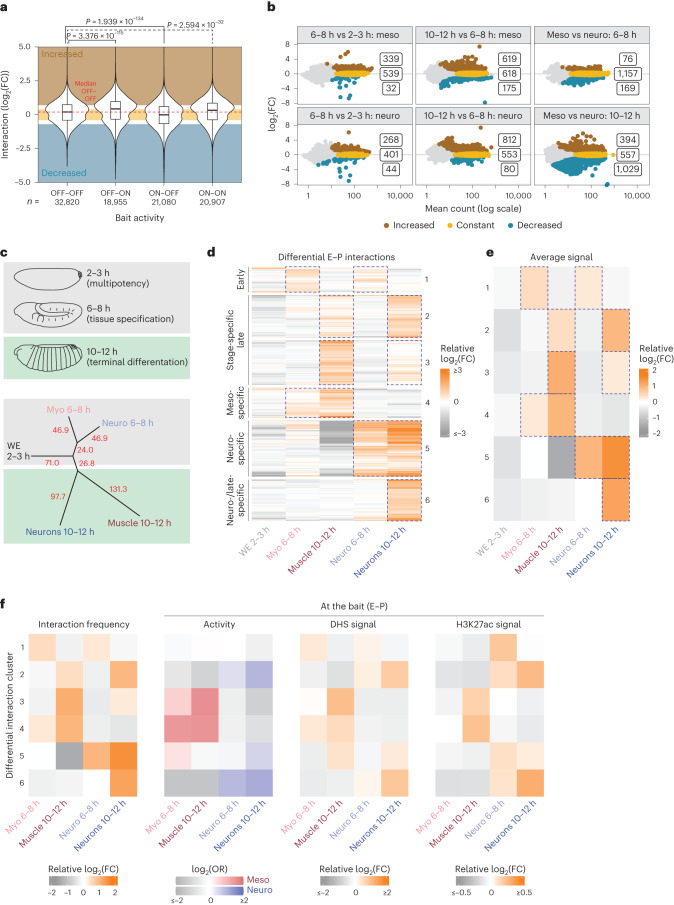
E–P proximity is tightly linked to activity during tissue differentiation but not during specification. **a**, Violin plot/boxplot showing changes in interaction frequencies (log_2_(FC)) of significant interacting regions for E/Ps (all E/P baits) changing in their activity between conditions (OFF–OFF, OFF–ON, ON–OFF and ON–ON). Number (*n*) of interacting fragments (CHiCAGO score ≥5) is indicated below. *P* value (above) from a nonparametric Wilcoxon test (two-sided) shows a significant concordant trend for increased or decreased interaction frequencies for E/P baits going from OFF–ON or ON–OFF. Boxplot, center = median; upper and lower bounds = interquartile range; whiskers = minimum and maximum. **b**, Scatter plot showing log_2_(FC) of interacting frequencies between conditions, with significantly increased (brown), decreased (turquoise) or invariant (yellow) interactions between the two conditions highlighted (numbers indicated). The majority of differential interactions are in comparisons involving 10–12 h tissues. **c**, Top, schematic representation of embryos at the relevant stages. Bottom, dendrogram displaying the distance (indicated by branch length) between the five developmental conditions based on their E/P interaction frequencies for all differential interactions (from **b**). Myoblasts and neuronal cells are closer to each other at 6–8 h than to their 10–12 h tissue counterparts (muscle or neurons). The two tissues are more divergent in their E–P interactions at 10–12 h (indicated by the long branch length). **d**, *K*-means clustering (*k* = 6) of all significant differential E/P interactions (from **b**), which increase (orange) or decrease (gray) relative to the sample average. Most differential interactions are higher at 10–12 h—clusters 2, 5 and 6 in the nervous system and clusters 3 and 4 in muscle. **e**, Average differential interaction frequency within respective clusters from **d**. Blue dotted outlines highlight conditions with high average interaction frequency in the respective clusters, for example, myo 6–8 h and neuro 6–8 h are outlined for cluster ‘Early’ in **d** and **e**. **f**, Comparison of average relative E/P interaction frequency per cluster (left, same as **e**) to E/P (bait) activity, shown as enrichment of in vivo annotated activity/expression (log(OR)), average DHS and H3K27ac ChIP–seq signal (log_2_(FC)) within each cluster in that condition. OR, odds ratio.

To assess the relationship between E–P proximity and activity, we categorized all E/P baits based on their dynamic activity in vivo from 2–3 h to 6–8 h or 6–8 h to 10–12 h in the muscle and nervous system (ON–OFF, OFF–ON, ON–ON and OFF–OFF). We first compared changes in E/P activity (Fig. 1b) to their global changes in interaction frequency, using all significantly interacting regions (both potentially instructive or permissive) across all conditions. This showed small, but highly significant, correlated changes (Fig. 2a). E/Ps that go from OFF-to-ON or ON-to-OFF have a concordant shift in interaction frequency going up or down compared to E/Ps with no change in activity (Fig. 2a and Supplementary Fig. 3a,b). There is therefore a global trend for E–P interactions to mirror changes in the E–P activity state, which may reflect new and/or a strengthening/weakening of existing interactions.

To assess this more formally, we identified a stringent set of 4,348 differential interactions (2,858 unique; 5% FDR (false discovery rate), >0.7 log_2_ fold change (FC) and CHiCAGO score ≥5) between any two conditions (Fig. 2b (brown and blue)) and 6,853 significantly constant (invariant) interactions (3,164 unique; Fig. 2b (yellow) and Supplementary Data 1; Methods). Most differential interactions occur in the transition from specification to terminal differentiation—68% (794/1,165) in myo and 74% (8,92/1,204) in neuro between 6–8 h and 10–12 h (Fig. 2b (brown and blue)). In comparison, there are fewer differential interactions between the multipotent blastoderm (2–3 h) and specification (6–8 h) stages (myo, 371 and neuro, 312) and more significantly constant interactions (Fig. 2b (left)). This is reminiscent of our previous observations comparing a later postgastrulation stage (3–4 h, stage 6/7) to 6–8 h by 4C-seq, where only ~6% of interacting regions changed^11^. However, we note differences in the interactions detected at the earlier blastoderm stage (2–3 h) compared to 6–8 h/10–12 h (Supplementary Note).

At the cell-fate specification stage, the number of significantly constant (invariant) interactions between myo and neuro at 6–8 h is surprisingly much greater (1,157; Fig. 2b (top-right)) than the number of differential interactions (76 up and 169 down = 245; Fig. 2b). This indicates that despite the activation of lineage-specific gene expression occurring during cell-fate specification, including the marker genes used to isolate these cells (Mef2 and Elav), many E–P topologies are very similar across cell types and are therefore generally not correlated to activity, fitting a more permissive mode of E–P regulation during specification.

Later, during terminal tissue differentiation (10–12 h), there is a substantial increase in the number of differential interactions (Fig. 2b (brown and blue, middle)). The majority are gains (78% (635/794) muscle and 91% (844/892) neurons; Fig. 2b), indicating that many E–P interactions are formed/strengthened during tissue differentiation and added to pre-existing topologies (Fig. 2b (middle)). As a consequence, the number of shared (invariant) interactions between the two tissues is lower at 10–12 h compared to 6–8 h—557 versus 1,157 (Fig. 2b (right, yellow)). Both results indicate more diversification in E–P interactions between tissues during differentiation (at 10–12 h) and more similarity during cell-fate specification.

In an independent analysis, we confirmed these results by taking the normalized interaction counts from all differential interactions (Methods) and constructing a dendrogram (Fig. 2c) and PCA (principal component analysis), Supplementary Fig. 3c). This analysis indicates more similarity in E–P interactions between cell types at 6–8 h (when cells are specified) than within a tissue across these two stages. For example, myoblasts are more similar to neuronal cells at 6–8 h in their E–P interactions than they are to differentiating muscle (10–12 h; Fig. 2c).

To directly assess the relationship between E–P proximity and activity, we clustered differential interactions based on their quantitative changes in interaction frequency and compared each cluster to the activity state of the E/P baits. The majority of differential interactions have high interaction frequency in one tissue (for example, clusters 3 and 4 in muscle and clusters 2, 5 and 6 in neuro; Fig. 2d,e (orange)) and lower quantitative signal (Fig. 2d,e (gray)) and lower CHiCAGO score (Supplementary Fig. 3d) in the other tissue/time point, indicating that differential interactions are robust and not due to thresholding effects. To compare E/P interactions to activity, we used the following three metrics of E/P activity (Fig. 2f): (1) the stringent in vivo annotation of the activity state of the E/Ps, (2) DHS^32^ and (3) H3K27ac data, which is tissue and stage matched. As the information on in vivo activity is binary, based on expression annotation (ON and OFF), we tested for significant enrichment of E/Ps active in the myogenic or neuronal tissues in each interaction cluster. To complement this, we assessed the quantitative DHS and H3K27ac signals at the E/P baits (Fig. 2f). Clusters with high E/P interaction frequencies in muscle at 10–12 h (clusters 3 and 4), for example, are enriched at enhancers and promoters active in muscle at 10–12 h, as seen by all three metrics (Fig. 2f). Similarly, clusters with high interaction frequencies in differentiating neurons 10–12 h (clusters 2, 5 and 6) are associated with E/Ps active in neurons at 10–12 h. This relationship between E/P proximity and E/P activity is more ambiguous at 6–8 h during cell-fate specification. Clusters 1 and 5, for example, have high interaction frequencies in myoblasts and neuronal cells at 6–8 h, respectively, but are not enriched in enhancers active at these stages (Fig. 2f (clusters 1 and 5)). This is exemplified at the *Delta* and *zfh1* loci, which are ON–ON at both 6–8 and 10–12 h in the nervous system (*Delta*) or both tissues (*zfh1*), yet have invariant interactions at 6–8 h, with differential interactions only at the later time point (Fig. 1e).

### Dynamic E–P loops are linked to dynamic regulatory features

**Fig. 3:**
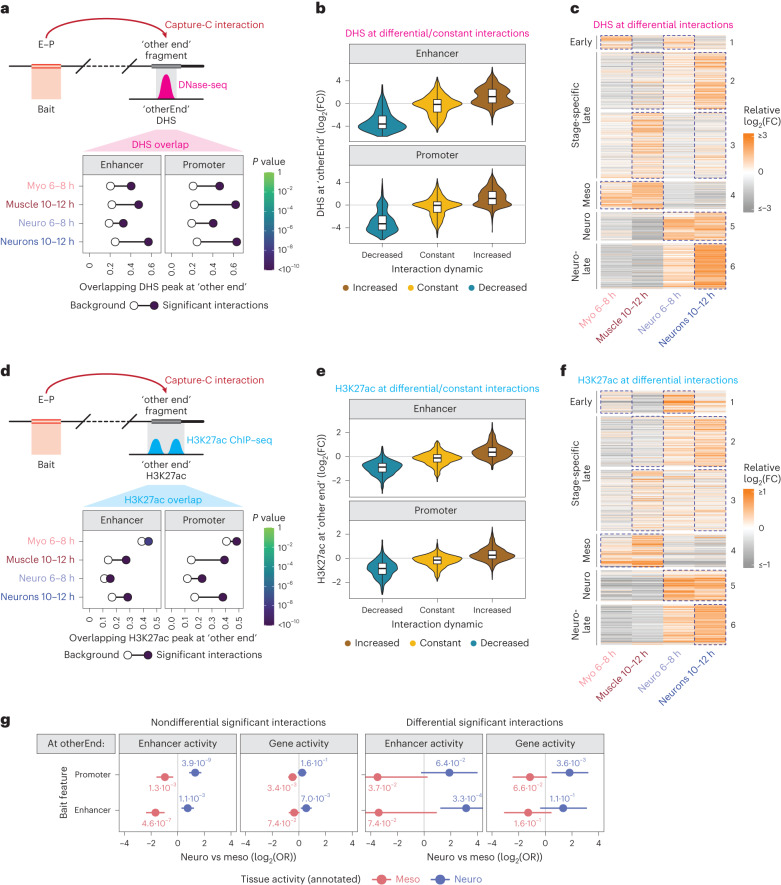
Dynamic E–P interactions are linked to dynamic regulatory features. **a**,**d**, Enrichment of tissue/stage-matched DHS (**a**) or H3K27ac peaks (**d**) in proximity to the ‘other end’ of all significant interactions (schematic, top). Bottom, frequency of DHS within 500 bp of the ‘other end’ in the respective condition, for all significant (filled circle) or nonsignificant (empty circle) interactions. DHS are more frequently at the ‘other end’ of interactions at 10–12 h, compared to 6–8 h (**a**). Color shade (filled circles) indicates *P* value (two-sided Fisher’s exact test). Enrichments for E or P baits are shown separately. **b**,**e**, Violin plots/boxplots showing quantitative changes in DHS signal (**b**) or H3K27ac ChIP–seq signal (**e**). The direction of change in DHS signal correlates with direction of change in interaction frequency (**b**). The direction of H3K27ac change correlates with changes in interaction frequency, especially at 10–12 h (**e**). log_2_(FC) at ‘other ends’ (<500 bp) of E/P Capture-C baits with decreased (turquoise), constant (yellow) or increased (brown) interactions across the same conditions (tissue or time). Boxplot, center = median; upper and lower bounds = interquartile range; whiskers = minimum and maximum. **c**,**f**, Relative DHS (**c**) or H3K27ac (**f**) quantitative signal at the ‘other end’ of differential interactions in six clusters in Fig. 2d. Higher DHS signal (**c**) and H3K27ac (**f**) signal mirror higher E/P interaction frequency in the respective clusters/conditions (blue dotted outline highlights relevant conditions for each cluster). **g**, E/P baits interact with enhancers or promoters (at the ‘other end’) that are active in the same tissue (either neuro or myo/muscle). Baits active in both tissues were excluded. *X* axis = enrichment (log_2_(OR)) of features active in the two tissues. Positive log_2_(OR) (red dot) indicates enrichment in E/P baits with neuro activity (relative to muscle), and negative value (blue dot) indicates enrichment in muscle activity (relative to neuro). Whiskers = 95% confidence interval. Left, enrichments for nondifferential E/P interactions. Right, enrichments for differential E/P interactions. E/P baits (both differential and constant) active in one tissue preferentially interact with genomic features active in the same tissue (*x* axis cut at −4/4, respectively).

Each interaction, both differential and constant, is defined by the two loop anchors—the bait (E–P) and their linked regions, named ‘other end’ from now on (Fig. 3a). The analysis mentioned above indicates that changes in the E/P bait activity are highly correlated with changes in proximity, predominantly during tissue differentiation (Fig. 2). Here we investigate if changes in proximity are also associated with changes in the activity of the element at the ‘other end’, focusing on the time points of specification (6–8 h) and differentiation (10–12 h), when the majority of differential interactions occur.

**Extended Data Fig. 2:**
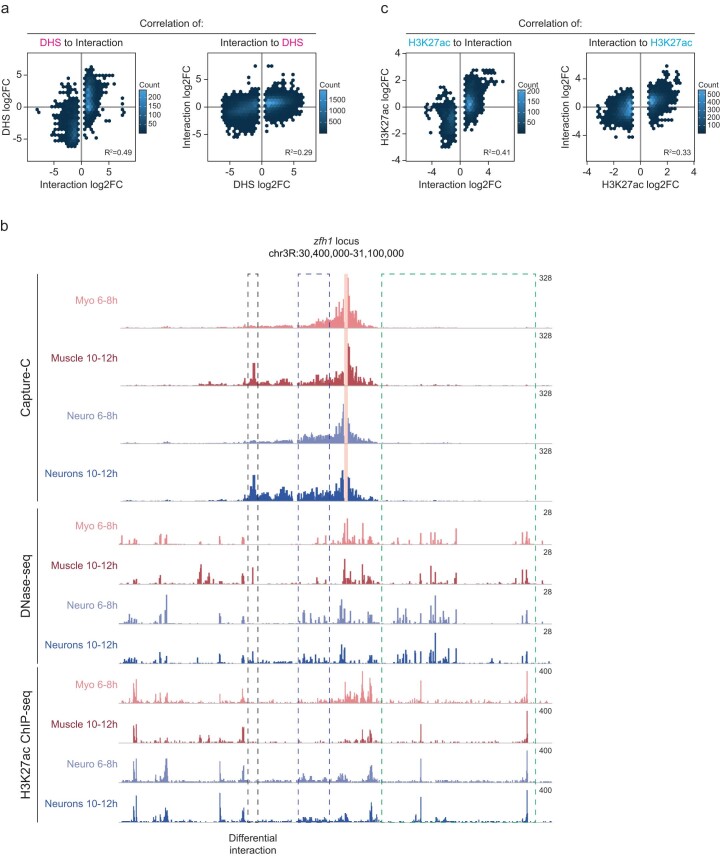
Differential E/P interactions are highly correlated with differential DHS, but not vice versa. **(a)** 2D density plot displaying DNase-seq (DHS) signal at the ‘other end’ with respect to interaction frequency at differential E/P interactions (left panel, DHS to interaction) or changes in interaction frequency with respect to changes in DNase-seq signal at differential DHS (right panel, Interaction to DHS). Increase or decrease of interaction frequency at differential E/P interactions is generally correlated with a concordant change in DHS (left panel). Changes in DHS signal (at differential DHS regions) are less correlated with changes in interaction frequencies (right panel). **(b)** Example locus showing coordinated and non-coordinated changes: normalized Capture-C counts at the *zfh1* promoter bait (highlighted in light pink) have a high-confidence interaction (leftmost rectangle) in both muscle and neurons at 10-12 h. Below, DNase-seq and H3K27ac ChIP–seq signal in matched conditions. The differential stage-specific E/P interaction overlaps a stage-specific DHS (left rectangle (black dashed outline)) in both conditions—and is an example of concordant changes in differential DHS and chromatin interactions. Other highly tissue-specific DHS or H3K27ac peaks (middle, blue dashed rectangle), which are in-between the bait (red bar) and the 10-12 h differential interaction, do not show a comparable increase in interaction frequency. Although these regions (middle blue dashed rectangle) are part of the *zfh1* regulatory landscape, the increase in, for example, DHS signal between Myo 6-8 h and Neuro 6-8 h is not mirrored by a concordant increase in interaction frequency. Other highly tissue- and/or stage-specific DHS and H3K27ac peaks to the right of the *zfh1* bait (right, green dashed rectangle) show very low interaction frequency and are not part of the *zfh1* regulatory landscape, again demonstrating that high DHS signal in the same tissue/time point is not necessarily linked to high E/P interaction frequency. **(c)** Similar to (**a**) for H3K27ac ChIP–seq signal, showing a general correlation between changes of interaction frequency and the underlying H3K27ac signal, while the reverse (changes in H3K27ac compared to interaction frequency) is less correlated.

We first assessed open chromatin using tissue- and stage-matched DHS^9^. Interacting regions (CHiCAGO score ≥5) are significantly enriched in DHS at the ‘other end’ (Fig. 3a and Supplementary Data 2). As DHS are highly enriched in TF binding, this indicates that developmental enhancers and promoters preferentially interact with regions bound by, or at least accessible to, TFs. Even more striking, the direction of change in E/P interactions is highly concordant with DHS changes, where increased interaction frequency is correlated with increased DHS signal in that tissue/time point and vice versa (Fig. 3b (brown and blue)). Conversely, constant/invariant interactions have little DHS changes between conditions (Fig. 3b (yellow)). This is mirrored in the clusters of differential interactions, which show highly correlated quantitative changes in DHS signal at the ‘other end’ in the same condition as the interactions are formed (Fig. 3c, compared to 2d). Notably, this is not the case in the other direction—a change in DHS signal does not necessarily lead to a change in E–P interaction frequency. Accessibility is much less correlated with Capture-C interactions (Extended Data Fig. 2a), indicating that the presence (or emergence) of a DHS is not always associated with the formation of a high-confidence (or differential) E/P interaction. There are many examples of interactions skipping a DHS (Extended Data Fig. 2b). This demonstrates that it is not merely accessibility, but the binding of specific factors to selected elements that regulates E–P interactions.

We next assessed if there were changes in activity at the ‘other end’, using H3K27ac as a proxy for active enhancers and promoters. To facilitate this, we performed tissue- and stage-specific chromatin immunoprecipitation followed by sequencing (ChIP–seq) for H3K27ac in matching tissues and stages (Fig. 1a). Similar to DHS, H3K27ac is generally enriched at the interacting ‘other end’, suggesting that E/Ps interact with regions that are likely active promoters or enhancers (Fig. 3d and Supplementary Data 3). Notably, differential E/P interactions have concordant changes in the H3K27ac signal (Fig. 3e)—increased or decreased E/P interactions are associated with increased or decreased H3K27ac signal at the ‘other end’ (Fig. 3e (brown and blue)). Conversely, constant/invariant interactions have little H3K27ac change (Fig. 3e (yellow)). This indicates that changes in E/P interactions are associated with changes in activity at the ‘other end’ (Fig. 3e), confirming our observations at the bait (Fig. 2f). Moreover, the levels of H3K27ac signal at the ‘other end’ generally reflect the changes in E/P interaction frequency (Fig. 3f compared to 2d).

A fraction of interacting ‘other ends’ overlap characterized embryonic enhancers or genes. Examining the activity of these elements shows that they preferentially interact with E/P baits that are active in the same tissue (Fig. 3g; Methods). For example, enhancers and genes active in myoblasts or muscle are enriched at the ‘other end’ of myogenically active baits (E/Ps; Fig. 3g (right, red)), conversely, neuronally active E/P baits interact with elements active in the nervous system (Fig. 3g (right, blue)). Dynamic (or tissue-specific) E/P interactions are therefore interacting with other elements (E/Ps) that are active in the same tissue/time point, providing further evidence that they are likely instructive loops with regulatory function, which we assess below. Interestingly, we note that such significant enrichments also hold true for all remaining nondifferential interactions, although to a lower extent (Fig. 3g (left)), suggesting that many invariant interactions (permissive loops) also likely have regulatory functions.

### Insulator binding at loop anchors cannot explain E–P loops

To explore how instructive (differential) or permissive (invariant) interactions are regulated, we first examined insulator proteins^41^. *Drosophila* has several insulator proteins (Supplementary Note)^42^; however, here we focused on three major ones, CTCF, BEAF-32 and Su(Hw), that bind to the majority of domain boundaries^36,43–45^ and are implicated in gene regulation^46–50^. To determine if they could regulate differential or invariant E/P interactions, we searched for (co-)occupancy at interacting regions. To facilitate this, we performed tissue- and stage-matched ChIP–seq (Fig. 1a), representing four conditions for three factors, each with biological replicates.

**Fig. 4:**
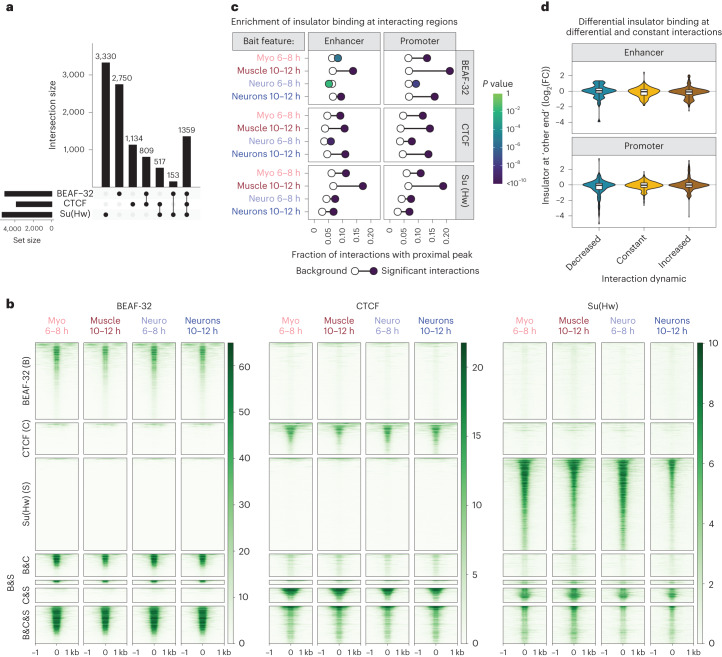
Insulator binding at loop anchors cannot explain E–P loops. **a**, UpSet plot of significant insulator ChIP–seq peaks across all conditions. **b**, Heatmaps of quantitative ChIP–seq signal for BEAF-32, CTCF and Su(Hw) in the indicated condition (above), ±1 kb around peak centers (0). Peaks were clustered based on the binding of BEAF-32, CTCF or Su(Hw) alone, or combined: B&C = BEAF-32 and CTCF; B&S = BEAF-32 and Su(Hw); C&S = CTCF and Su(Hw); B&C&S = all three factors. **c**, Enrichment analysis of all insulator peaks in proximity to significantly interacting regions. The frequency of insulator peaks in proximity (<500 bp) to the ‘other end’ of significant (filled circle) and nonsignificant (empty circles) interactions in the respective samples. Color shade of filled circles denotes the *P* value (two-sided Fisher’s exact test). Data for the enhancer and promoter baits are shown separately. CTCF and Su(Hw) are enriched at the ‘other end(s)’ of both E/P interactions, whereas BEAF-32 is more enriched at interacting regions of promoter baits. **d**, Violin plots/boxplots showing quantitative changes in insulator binding (log_2_(FC) of ChIP–seq signal) at ‘other end(s)’ (<500 bp) of Capture-C E/P baits with decreased (turquoise), constant (yellow) or increased (brown) interactions, across the matched conditions (tissue/time). Changes in E/P interactions are globally not correlated with changes in insulator binding. Boxplot, center = median; upper and lower bounds = interquartile range; whiskers = minimum and maximum.

Each factor binds to thousands of sites (FDR 0.05) in one or more condition (Fig. 4a; Methods), with 2,838 regions bound by two or more insulator proteins (within 50 bp; Fig. 4a,b and Supplementary Data 4–6; Methods). Although almost half (44% (4,429/10,052)) of all insulator peaks have a significant change (FDR 0.05 and >0.7 log_2_(FC)) in binding between conditions (Supplementary Fig. 4a; Methods), many of these regions remain bound by another insulator protein. Examining the quantitative signal indicates that the binding of any factor is remarkably similar across time and tissues (Fig. 4b). Su(Hw) has the most occupancy changes—1,959 peaks have reduced binding at the later time point (Supplementary Fig. 4b). Differential insulator peaks are generally located within TADs, whereas constant (tissue/stage invariant) peaks are enriched at TAD boundaries and typically include all three proteins (Supplementary Fig. 4c–e).

Insulator binding is generally enriched at the ‘other end’ (within 500 bp) of E/P interactions (Methods). However, 74–79% of significant (and 56–70% of high-confidence) interacting regions are not directly bound by any of these insulators (Fig. 4c). Across the entire dataset, only 34% (2,965/8,778) of enhancers and 39% (5,443/13,933) of promoter high-confidence interactions are bound by one or more insulators in the same tissue/time point.

We found a small number of cases with correlated changes in differential insulator binding and differential E/P interactions. These usually involve a tissue-specific gain of CTCF and loss in some cases of Su(Hw), for example, *robo3* (Supplementary Fig. 4f), similar to the *Ubx* locus^51^. However, globally, changes in E/P interactions are not correlated with changes in insulator binding at the loop anchor at the matched time/tissue (Fig. 4d). This is in sharp contrast to changes in DHS and H3K27ac signal at the ‘other end’, which are both highly correlated and concordant with changes in E/P interactions (compare Fig. 4d with Fig. 3b,e). Although we cannot exclude that some E–P interactions might be regulated by insulator binding, the majority appear to be regulated by other factors.

**Extended Data Fig. 3:**
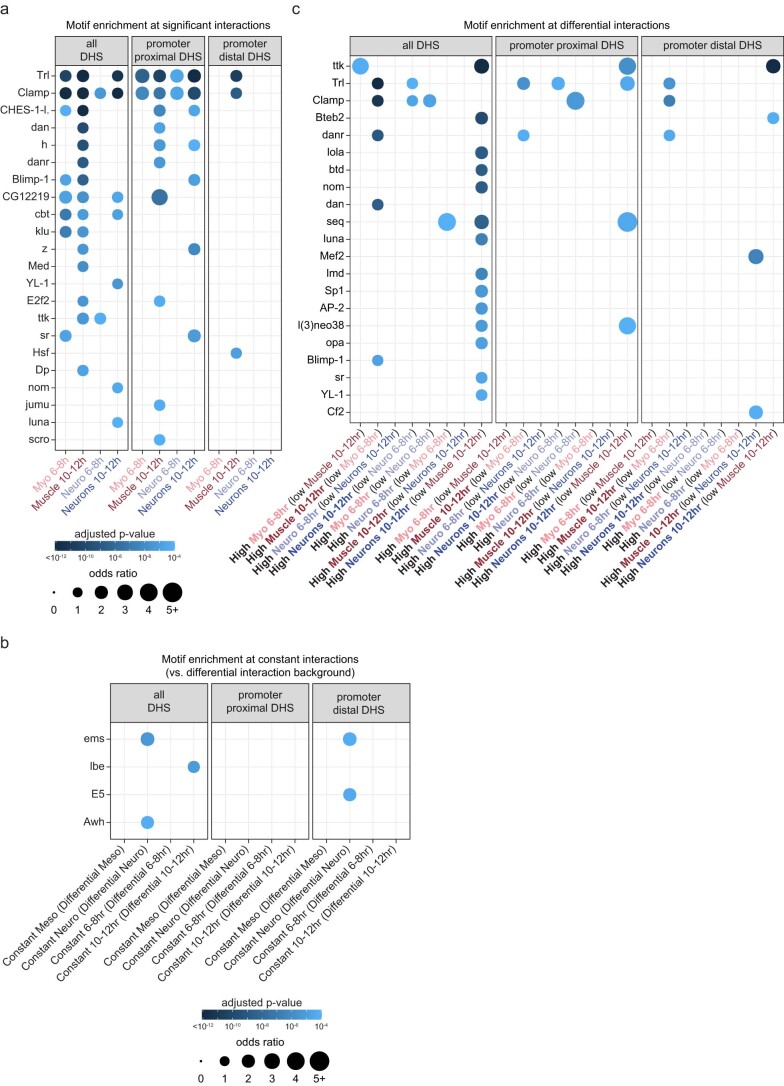
Motif enrichment at E/P loops—instructive tissue-specific loops are enriched in motifs for tissue-specific transcription factors. **(a)** Identification of potential factors involved in the formation of E/P loops. Tissue- and stage-matched DHS (from ref. ^32^) were divided into two groups, a test set in proximity (<500 bp) to all significant interactions and a control set (composed of a non-overlapping DHS set that is in proximity (<500 bp) to non-significant interactions). Enrichment of *Drosophila melanogaster* transcription factor motifs (from CIS-BP) in the test DHS relative to control DHS (Methods). Plot shows motifs enriched in the indicated sample using an adjusted p-value cutoff of 1 × 10^−^^4^. **(b)** Motif enrichment comparing constant interactions to differential interactions (using DHS underlying differential interactions as the background set for enrichment calculation). Plots show motifs enriched in the indicated sample using an adjusted p-value cutoff of 1 × 10^−4^. All four factors have multiple PWMs, which are variants on TAATTA sequence, suggesting that this enrichment likely comes from the same factor. **(c)** Motif enrichment at differential interactions. DHS were divided into three groups based on their proximity (<500 bp) to increased, decreased or “other” (non-increased and non-decreased) E/P interacting regions characterized in the same tissue/time condition. Enrichment of *Drosophila* transcription factor motifs (from CIS-BP) in either the increased or decreased DHS, relative to other DHS in the same condition, was carried out using the AME tool (doi: 10.1186/1471-2105-11-165). In (**a**), (**b**) and (**c**) for the background, only DHS >10 kb and <250 kb from the bait were considered, and enrichments for all, promoter proximal and promoter distal DHS (≥500 bp) are shown separately. p-values in (**a**), (**b**) and (**c**) were calculated using a one-sided Fisher exact test.

To identify potential regulators of E–P interactions, we searched for motifs enriched within the underlying DHS from matched tissues and stages (Methods). First, searching within all significantly interacting regions (using noninteracting tissue/stage-matched DHS as background) identified 20 motifs (Extended Data Fig. 3a; adjusted *P* < 1 × 10^−^^4^; Methods). This includes motifs for factors known to have a role in E–P communication or chromatin topology, for example, Trl/GAF^52–54^ and Clamp^55,56^, as well as factors that have not been implicated to date (Extended Data Fig. 3a). Motifs for insulator proteins were not enriched, again indicating that they are not major regulators of E–P interactions (at least not directly at the loop anchors).

To determine if there are distinguishing motifs between permissive versus instructive interactions, we directly compared DHS underlying constant (as a proxy for permissive) versus differential interactions (Methods), which identified four motifs (Extended Data Fig. 3b; adjusted *P* < 1 × 10^−4^). None of these proteins have known roles in chromatin topology and all four are homeobox TFs with similar motifs, and therefore likely represent the same factor. Differential interactions (compared to matched DHS of nondifferential interactions) identified seven motifs, which include several TFs essential for the respective tissue’s development, including Mef2 (ref. ^57^), enriched at muscle (compared to neuron), and l(3)neo38 (ref. ^32^) enriched at neuronal (compared to muscle; Extended Data Fig. 3c). These enrichments are against a background of tissue-matched DHS and suggest that these lineage-specific TFs may have a role in regulating instructive loop formation, either directly or via activation of developmental enhancers, which then form a loop.

### Concordant chromatin changes can reveal functional E–P pairs

**Fig. 5:**
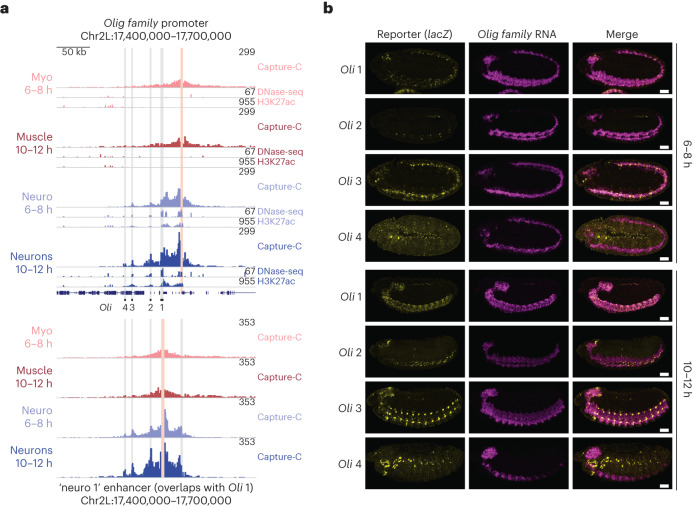
Concordant changes in E/P interactions and chromatin features reveal functional E–P pairs. **a**, Top, normalized Capture-C, DNase-seq and H3K27ac ChIP–seq signal at the *Olig family* (*Oli*) locus in four conditions. Vertical light pink bar indicates the bait (*Oli* promoter), and gray bars indicate the position of interacting regions tested for enhancer activity. Bottom, reciprocal Capture-C from the neuro 1 (*Oli* 1) enhancer (bait = vertical light pink bar). Normalized Capture-C counts in four different conditions. Significant interactions between neuro 1 and the *Oli* promoter and other elements are indicated by gray bars. **b**, Double FISH of embryos for four transgenes testing enhancer activity of genomic regions *Oli* 1–4 at the indicated stages. Yellow indicates reporter RNA (*lacZ*) and magenta indicates *Oli* RNA. Regions *Oli* 1–3 function as enhancers overlapping part of *Oli* expression at 10–12 h. Scale bars = 50 µm.

**Extended Data Fig. 4:**
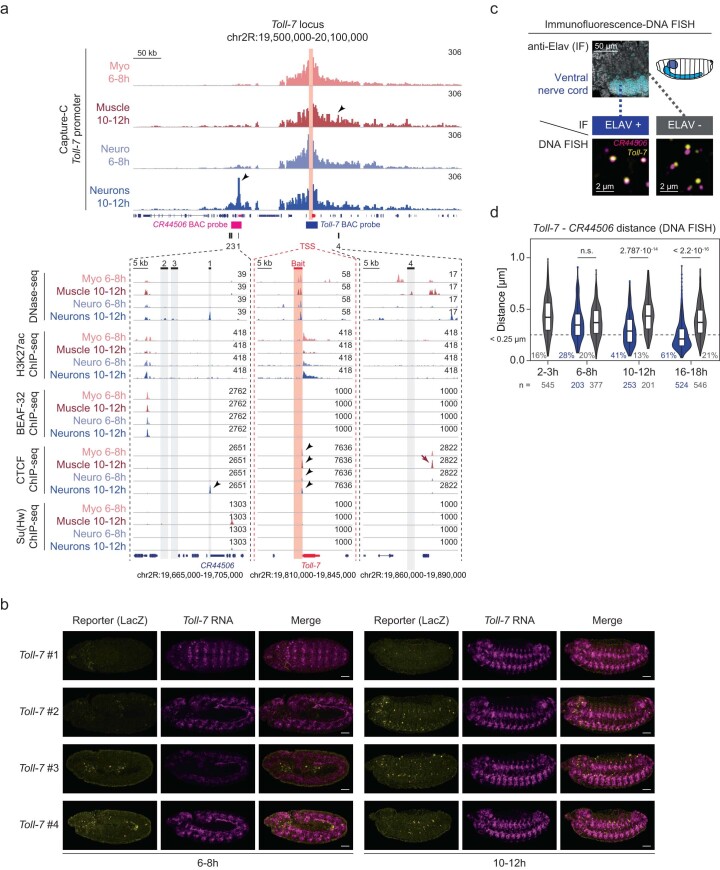
Differential Capture-C interactions at the *Toll-7* locus represent functional enhancer elements. **(a)** Upper: normalized Capture-C signal at the *Toll-7* locus in 4 conditions. Vertical light pink bar = bait (*Toll-7* promoter), gray bars (zoom-in) = position of interacting regions tested for enhancer activity. Genomic location of BAC probes used for DNA FISH (blue, magenta rectangles) and genomic regions tested in transgenic enhancer assays (labeled 1-4) are shown below. Lower: zoom-in showing DNase-seq (DHS), H3K27ac and insulator ChIP signal in the 4 tested elements *Toll-7* 1-4 and *Toll-7* gene. Differential interaction between *Toll-7* promoter and *CR44506* at 10-12 h in neurons is accompanied by differential CTCF binding in neurons at 10-12 h (black arrowhead). Muscle-specific *Toll-7* promoter and *Toll-7*4 coincide with adjacent muscle-specific CTCF binding (red arrow). Perhaps differential insulator binding plays a role in differential E/P interactions at this locus. **(b)** Double fluorescence *in situ* hybridization of transgenic embryos testing *Toll-7* 1-4 for enhancer activity. Yellow = reporter (*lacZ*), magenta = *Toll-7* RNA. *Toll-7* 2-4 have sporadic enhancer activity in a small subset of cells (scale bars = 50 µm). **(c)** Immunofluorescence (IF)-DNA FISH: above, IF signal of Elav expression in the ventral nerve cord (false-colored in cyan, DAPI in gray) of stage 16 embryo (lateral view, single optical section, scale bar = 50 µm). Below, DNA FISH (yellow = *Toll-7*, magenta = *CR44506*, BAC probes position indicated in (**a**). Zoom-in of Elav+ (lower left) or Elav− (lower right) region (maximum projection from deconvolved image stacks, scale bars = 2 µm). 3D distance between *Toll-7*-*CR44506* was measured in neuronal (Elav+) and adjacent non-neuronal (Elav−) tissue within the same embryos. **(d)** Violin plot/boxplot of DNA FISH distance between *Toll-7* and *CR44506* in neuronal (blue = Elav+) and non-neuronal (gray = Elav−) tissue. Dashed line = 250 nm. Percentage with distances <250 nm, number (n) of nuclei measured indicated underneath. The two loci are significantly closer in neuronal compared to non-neuronal cells at 10-12 h and 16-18 h. P-values from Kolmogorov-Smirnov test (two-sided). Boxplot: center = median, upper/lower bounds = first/third quartiles, whiskers = lowest/highest at min/max 1.5 interquartile range, dots = outliers plotted individually.

**Extended Data Fig. 5:**
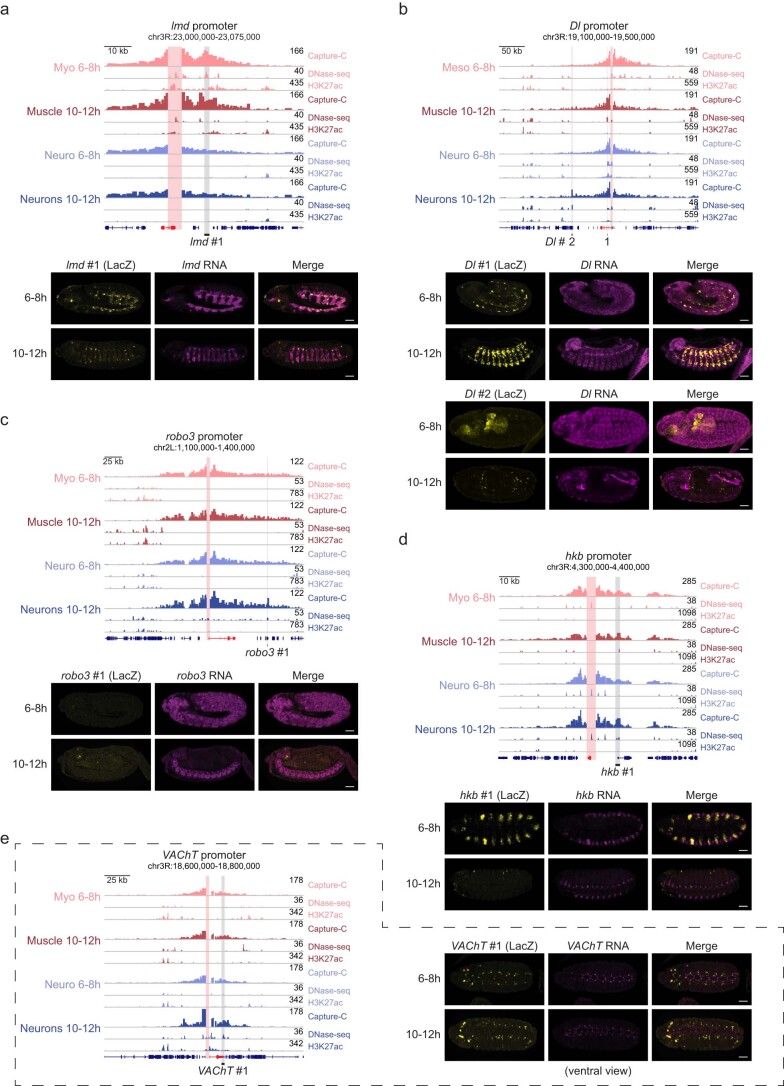
Tissue-specific promoter interacting regions are often enhancers active in that tissue. **(a)** Upper: normalized Capture-C, DNase-seq and H3K27ac ChIP–seq signal at the *lame duck* (*lmd*) locus in 4 different conditions. Vertical red bar = *lmd* promoter (bait), gray bars = tested interacting region (*lmd* 1) in transgenic embryos. Lower: RNA *in situ* hybridization in transgenic embryos for the reporter gene (yellow, *lacZ*) and the *lmd* gene (magenta) at the indicated stages, for lmd 1. **(b**–**e)** As in (**a**) for *Delta* (*Dl*) interacting regions (**b**), *roundabout 3* (*robo3*) region (**c**), *huckebein* (*hkb*) region (**d**) and *vesicular acetylcholine transporter* (*VAChT*) region (**e**) (scale bars = 50 µm). Five of the tested regions (5/19) either had enhancer activity that did not match the interacting gene’s expression (VAChT **(e)**, *Oli* 4 (Fig. 5)), or did not match the tissue-specific interactions (*Dl* 2, **(b)**), or had no enhancer activity (*Dop1R1*, *Toll-7* 1 (Extended Data Fig. 4b)) (Supplementary Table 1). For example, the interacting region with the *VAChT* promoter **(e)** had enhancer activity in the central nervous system, but curiously only in cells adjacent to the gene’s expression. The *Dl* 2 interacting region **(b)** has activity overlapping the *Dl* gene’s expression in the endoderm and visceral muscle, but this does not match the predominantly neuronal-specific interaction between the *Dl* 2 enhancer and the *Dl* promoter at 10-12 h **(b)**. Some interacting elements might be bystander interactions in a gene dense and/or very compact locus (that is, cases where the enhancer’s activity does not match the interacting gene’s expression) or might serve a different regulatory function (that is, cases where the element does not function as an enhancer at all for example *Toll-7* 1).

Given the general concordance between tissue-specific changes in E/P interactions and activity at both the bait (Fig. 2) and ‘other end’ (Fig. 3), we reasoned that correlated changes in interactions, accessibility and H3K27ac could identify functional E–P pairs. To assess this, we selected 12 loci (promoter baits) with different properties and determined if their interacting regions (19 in total) function as enhancers in vivo and recapitulate part of the gene’s expression (Fig. 5, Extended Data Figs. 4 and 5 and Supplementary Table 1).

The *Olig family* (*Oli*) gene is expressed in neurons and required for motoneuron axon pathfinding^58^. The *Oli* promoter interacts with a number of genomic regions specifically in neuronal cells—*Oli* 1–3 from 6–8 h and a more distal region (*Oli* 4) at 10–12 h (Fig. 5a (upper)). *Oli* 1 (called ‘neuro 1’, based on tissue-specific accessibility^24^) was included in our enhancer baits, and we detect a reciprocal interaction with the *Oli* promoter and the other three putative regulatory elements (*Oli* 2–4), confirming these interactions (Fig. 5a (lower)). All regions (except the more distal *Oli* 4) have a DHS and H2K27ac peak in neuronal cells and not muscle (Fig. 5a) and are therefore examples of tissue- and stage-specific interactions only in the tissue where the gene is expressed. We tested all four regions for enhancer activity in transgenic embryos (Methods). Three of the four regions have neuronal enhancer activity overlapping *Oli* expression at the appropriate stage, confirming that these regions are neuronal enhancers and suggesting that their promoter interaction is instructive (Fig. 5b). The most distal region (*Oli* 4) showed no overlap with *Oli* expression and is also the region with no H3K27ac signal.

In the *Toll-7* locus, we tested four promoter-interacting regions, three of which have neuronal (*Toll-7* 1–3) and one muscle-specific (*Toll-7* 4) DHS at 10–12 h^32^; however, none have H3K27ac peaks (Extended Data Fig. 4a). Three regions (*Toll-7* 2–4) showed very weak enhancer activity overlapping *Toll-7* expression in a small subset of cells (Extended Data Fig. 4a,b). The fourth, *Toll-7* 1, which has the most significant differential interaction, has no enhancer activity and overlaps the promoter of a long noncoding RNA (lncRNA), *CR44506*. We confirmed that this ~130 kb *Toll-7-CR44506* loop is specific to the nervous system, and differentiation stage, by DNA fluorescence in situ hybridization (FISH; Extended Data Fig. 4c,d). Some differential interactions at other loci also involve lncRNA genes (Supplementary Fig. 5).

In total, of the 19 interacting elements tested in vivo (Supplementary Table 1), *Oli* (4 test regions), *Toll-7* (4 test regions), *Dl* (2 test regions), *lmd*, *bap*, *tin*, *robo3*, *hkb*, *VAChT*, *danr*, *chinmo*, *Dop1R1*, 14 (74%) showed enhancer activity in the correct cell type, and at least partially overlaps the expression of the gene (for example, *lmd* in a subset of somatic muscle, *Dl* 1 in late muscle; Extended Data Fig. 5a,b). For some elements, the enhancer activity was weak and limited to a small subset of cells, for example, the *robo3* in the brain (Extended Data Fig. 5c), while others were very transient in the ‘correct’ tissue, for example, *hkb* (Extended Data Fig. 5d).

These results indicate that combining tissue-specific changes in E–P proximity with concordant changes in chromatin accessibility and/or H3K27ac is generally a good indicator of functional E–P pairs. However, this is not always the case (seen here for 26% of tested cases), and it is not obvious why this works so well for some loci and not others.

## Discussion

Our findings provide one explanation for why different relationships between E–P proximity and activity may have been observed, which is the developmental state of the cell. During cell-fate specification (and earlier), E–P interactions are surprisingly similar (at least between myoblasts and neurons), although these cell types have many differences in their enhancer activity and gene expression. As a result, their E–P proximity is less correlated with activity and rather seems permissive, ready for activation. At later embryonic stages, during terminal tissue differentiation, there is a switch to more instructive E–P topologies, when many new, often more distal interactions emerge, which are formed on top of pre-existing landscapes. Therefore, at the stages of tissue differentiation, *Drosophila* has many more changes in E–P contacts than previously observed (where studies focused on early stages) and is very consistent with recent observations in differentiated mouse tissues^59^. The use of more instructive E–P interactions therefore appears to be an ancient feature of gene regulation during tissue differentiation.

In the context of embryogenesis, it is interesting to speculate why different stages would use different types of E–P topologies. Genes expressed during zygotic genome activation and early blastoderm tend to be short and intronless, compared to genes expressed in differentiated tissues, which are often long with complex alternative splicing. Perhaps the three-dimensional features of E–P landscapes follow a similar logic. Early in embryogenesis, genes rely on permissive topologies where E–Ps act within predefined more proximal windows to support the very rapid changes in early embryogenesis. At mid-embryogenesis, highly similar E–P topologies between different cell types during specification may facilitate plasticity, which is essential for trans-differentiation of cell types. At even later stages, E–P topologies diverge between tissues, which might enable developmental lockdown during terminal tissue differentiation.

## Methods

### Ethics statement

No ethical approval or guidance is required. *Drosophila melanogaster* is an invertebrate and as such is not considered an animal for ethical approval. All experiments were on wild-type reference strain (Oregon R) embryos of mixed sex at the indicated time points.

### Resources used for enhancer and gene activity

The regulatory elements (both enhancers and promoters) targeted for capture (baits) were hand-selected from (1) curated databases of in vivo validated enhancers in transgenic embryos (that is, FlyEnhancers^29^, RedFly^30^ and CAD4 (ref. ^28^)) and (2) a curated database of in situ hybridization patterns for thousands of genes (BDGP (Berkeley *Drosophila* Genome Project) in situ database^31^). The expression patterns were mapped back to 1 of 16 higher-order classifications—central nervous system (CNS), ectoderm/epidermis (EctEpi), foregut (FoGut), endoderm and midgut (EndoMidgut), hindgut (HiGut), mesoderm/muscle (MesoMuscle), salivary gland (SalGl), tracheal system (Tracheal), stomatogastric nervous system (SNS), endocrine system and heart (EndocrineHeart), blood and fat (BloodFat), imaginal primordia (ImagPr), peripheral nervous system (PNS), visual primordia organ system (VisualPr), pole cells and germ cells of the gonad (Pole/Germ cell) and extraembryonic tissues (Extraemb). This mapping was performed using the annotation provided by ref. ^60^. Terms with missing higher-order mappings were mapped manually (provided in Supplementary Table 1). To classify features as active in mesoderm and its derived myoblasts and muscle, we searched for activity in MesoMuscle or EndocrineHeart. To classify features as active in neuronal tissue, we searched for activity in either CNS or PNS. Notably, we manually checked the activity of all selected enhancers and genes by visual inspection of available images to confirm their tissues and stage of activity.

For the BDGP data, gene IDs were converted to release 13 of the Dm6 genome (dmel_r6.13) using the FlyBase ID ‘Validator’ tool (FlyBase^61^). Genes that did not have a one-to-one annotation transposition were discarded. Gene promoters were defined as 500 bp upstream and 100 bp downstream of the genes’ first TSS. The coordinates of the FlyEnhancer enhancers were converted from Dm3 to Dm6 using the UCSC LiftOver tool. Intersects between the bait/otherEnd and the promoter/enhancers were defined as an overlap of one or more base pairs.

### Isolation of cell-type-specific nuclei for Capture-C and ChIP–seq

Nuclei were purified from the myogenic and neurogenic lineages from 6–8 h and 10–12 h staged embryos by fluorescence-activated nuclei sorting (FANS) from fixed embryos using our previously optimized BiTS protocol^32,62^ and described in more detail in the Supplementary Information. A rabbit anti-Mef2 antibody (1:75–1:100) was used to mark myogenic mesoderm and muscle derivatives, and a monoclonal mouse anti-Elav antibody (1:40) was used to mark neuronal cells (Supplementary Table 1). Only collection tubes with >95% purity for the gated population were used (most exhibited >98% purity). Sorted nuclei were pelleted by centrifugation at 3,200*g* in a swing-out rotor for 15 min at 4 °C and transferred in a small amount of PBT to 1.5-ml LoBind tubes and pelleted again at 3,200*g* in a tabletop centrifuge for 15 min at 4 °C. The nuclear pellet was snap-frozen at −80 °C for later use in Capture-C or ChIP–seq experiments.

### Capture-C in specific tissues and stages

To provide a high-resolution view of E–P interactions, a 4 bp cutter (DpnII) was used for the Capture-C, providing a theoretical resolution of ~254 bp, and all libraries were sequenced to a high sequencing depth. To ensure enough biological complexity to capture interactions for all regulatory elements, 100 million sorted snap-frozen nuclei were used per replicate (per condition) for each bait library (~350 baits per library). Capture-C was performed on two independent biological replicates per tissue and time point (five sample conditions, two bait pools), resulting in the following 20 Capture-C datasets: 4 WEs (2–3 h), 4 myoblasts (myo; 6–8 h), 4 muscle (10–12 h), 4 neuro (6–8 h) and 4 neurons (10–12 h). At the same time, we also actively sorted non-meso and non-neuro (NM/NN) nuclei (Mef2^−^/Elav^−^) from the same embryos, representing a heterogenous mixture of ectoderm and endodermal tissues at both 6–8 h and 10–12 h, and include the raw data for this set of 12 Capture-C datasets (4 NM/NN (6–8 h) and 8 NM/NN (10–12 h)) as a resource in the public repository ArrayExpress submission (accessions: E-MTAB-9310).

A detailed Capture-C protocol is available in the Supplementary Information. In brief, frozen fixed sorted nuclei were resuspended in ice-cold permeabilization buffer (10 mM Tris–HCl (pH 8.0), 10 mM NaCl, 0.2% (vol/vol) NP-40 supplemented with complete protease inhibitors without EDTA) and incubated on a nutator for 30 min at 4 °C (100 mio. in a total of 50 ml). After incubation, nuclei were pelleted at 600*g* at 4 °C for 10 min, the supernatant aspirated and the nuclei resuspended in 800 µl ice-cold 1.2× DpnII buffer (NEB) and mixed by inversion (25 mio. per reaction). Nuclei were pelleted again at 600*g* at 4 °C for 10 min and resuspended in 400 µl 1.2× DpnII buffer with 6 µl 20% (wt/vol) SDS (final concentration ~0.3%). The samples were incubated for 1 h at 37 °C in a thermomixer at 950 r.p.m. After incubation, 40 µl (20%; vol/vol) Triton-X-100 was added (final concentration ~1.8%) and incubated for 1 h at 37 °C at 950 r.p.m. in a thermomixer. Two aliquots of 15 µl (750 U) DpnII (NEB, 50,000 U ml^−1^) were added per sample several hours apart and digested for 16–24 h at 37 °C at 950 r.p.m. in a thermomixer. Nuclei were pelleted at 600*g* at 4 °C for 10 min and resuspended in ligation buffer (66 mM Tris–HCl (pH 7.5), 5 mM MgCl_2_, 5 mM DTT, 1 mM ATP, 100 ng µl^−1^ BSA (NEB), 240 U T4 DNA Ligase (Thermo Fisher Scientific)). Samples were incubated for ≥6 h at 16 °C followed by proteinase K digest, decrosslinking, RNase treatment and DNA precipitation using ethanol (Methods). For fragmentation, up to 6 µg of DNA, in a total volume of 120 µl, was sonicated to ~200 bp using a Covaris S2 sonicator. Sonicated samples were transferred to a new 0.5-ml tube and the DNA size was selected using SPRIselect beads (1.8× volume) and recovered in ~60 µl water. A total of 1 µg sonicated, size-selected DNA was used for library preparation per sample using the NEBNext Ultra DNA Library Prep Kit II, following the manufacturer’s instructions, followed by size-selection using one volume of SPRIselect beads (Methods). Multiplexed and pooled libraries were subjected to two rounds of oligo capture using the Nimblegen SeqCap EZ Hybridization and wash kit, following the manufacturer’s instructions (Methods). Each round of oligo capture was followed by PCR amplification using KAPA HiFi HotStart ReadyMix (Methods). Eluted DNA was analyzed using Qubit dsDNA BR Assay Kit and Bioanalyzer and used for sequencing. Capture-C libraries were sequenced with 150 bp paired-end reads using Illumina HiSeq2000 (software HCS v2.2.68) and HiSeq4000 (HCS v3.4.0) platforms at the EMBL Genomics Core Facility.

### Tissue-specific ChIP–seq on insulator proteins and H3K27ac

Similar to the Capture-C, purified myogenic and neurogenic nuclei at 6–8 h and 10–12 h were obtained by FANS as described previously^32,62^. ChIP–seq was performed as described in refs. ^62,63^ and in the Supplementary Information, using the following antibodies: rabbit anti-CTCF, goat anti-Su(Hw) and rabbit anti-H3K27ac (Supplementary Table 1). We used 2.5 µg chromatin for incubations with 1:900 anti-CTCF antibody, 2 µg chromatin and 1:300 anti-Su(Hw) antibody, 1 µg chromatin with 1:900 anti-BEAF antibody and 2 µg chromatin with 1:900 anti-H3K27ac antibody. The quality of the libraries was assessed on a Bioanalyzer (Agilent Technologies), and libraries displayed a peak around 350–600 bp. For each ChIP, at least two completely independent biological replicates were performed. ChIP–seq libraries were sequenced with 75 bp paired-end reads using the Illumina NextSeq 500 platform (NSS v2.2.0) at the EMBL Genomics Core Facility.

### Generation of transgenic lines for enhancer reporter assays

Interacting regions selected for in vivo testing for enhancer activity in transgenic embryos were amplified from genomic DNA from a reference *Drosophila* strain by PCR (primers listed in Supplementary Table 1). Each region was cloned, using In-Fusion cloning (Takara Bio) or Snap Assembly Master Mix (Takara Bio), into the pattB-Hsp70-LacZ plasmid (linearized using XbaI) upstream of a *Hsp70* minimal promoter driving expression of a *lacZ* reporter gene. Primers were designed using the In-Fusion Cloning Primer Design Tool v1.0 (Takara Bio) and are listed in Supplementary Table 1. All constructs were injected into embryos containing the attP landing site M(3×P3-RFP.attP′)ZH-51C via PhiC31 integrase insertion, yielding integration at chromosomal position 51C1 (ref. ^64^; Fly line: y w M(eGFP.vas-int.Dm)Zh-2A; M(RFP.attP)ZH-51C).

### Capture-C data analyses: defining high-confidence interactions

The Capture-C FASTQ files were aligned using HiCUP^65^ (version 0.6.1) with default parameters with --shortest and --longest set at 75 and 1,200, respectively. HiCUP used Bowtie2 (ref. ^66^; version 2.3.5) as the aligner and the Dm6 genome. The output BAM file generated by HiCUP was converted to the CHiCAGO input using the bam2chicago.sh script supplied along with the CHiCAGO package^33^ (version 1.14.0). CHiCAGO design files were generated with the following parameters: minFragLen=75, maxFragLen=1200, maxLBrownEst=75000, binsize=1500, removeb2b=True and removeadjacent=True. CHiCAGO was run using the design files, and significant interactions above a background distance decay were defined using a score threshold of ≥5. This identified 52,980 significant interactions across all baits in one or more of the five conditions (2–3 h WE, 6–8 h myo, 10–12 h muscle, 6–8 h neuro and 10–12 h neurons), which represents 35,693 unique interactions (Supplementary Data 1). Chromatin conformation capture techniques (including Capture-C, used here) typically capture bystander interacting fragments around the biological interacting region, for example, not only a fragment containing an enhancer or promoter but also the neighboring fragments around it. To remove such bystander interactions, we postfiltered the CHiCAGO-defined (≥5.0) significant interactions, retaining interactions that overlap a fragment with biological activity that is the fragment overlying a bound region, based on the presence of a significant DHS peak in the equivalent stage and tissue, obtained from ref. ^32^. The overlap of significant interacting regions with DHS peaks was lowest at 2–3 h compared to the other conditions. This may reflect a lower quality of the Capture-C data, although the QC metrics were not dramatically different from the other time points (Supplementary Fig. 1c–f), or alternatively the lower number of DHS peaks in the ref. ^32^ dataset at the overlapping 2–4 h time-window—which contains 7,423 DHS at 2–4 h, while the other four conditions have more than 18,000 peaks (Fig. 1b (ref. ^32^)). We also removed interacting regions very proximal (<2 kb) or distal (>10 Mb) to the baits (Methods). This defined a high-confidence set of 24,012 E–P interacting regions at one or more conditions (Fig. 1c,d and Supplementary Data 1), giving an overview of E–P interactions. A more quantitative assessment of the dynamic and tissue-specific changes in E–P interactions is provided in Fig. 2 (using DESeq2), using all interacting fragments.

Of the 637 bait regions captured across the two libraries, 51 were not analyzed further due to the redundant targeting of a genomic feature better captured by another set of probes. A further three bait regions were discarded due to inefficient capture efficiency, as seen by the failure to capture any significant interactions in any of the experimental samples. This postfiltering left 583 regulatory regions with high-quality captures. Of these, 26 regions were captured in both bait libraries allowing us to better determine the reproducibility of the data (Supplementary Fig. 1g,h).

### Identifying differential and constant Capture-C interactions

To identify significantly differential and constant (invariant) interactions (Fig. 2b), fragment counts and CHiCAGO scores were extracted from the CHiCAGO output. Only *cis* interactions involving the major chromosomes (chr), chr2L, chr2R, chr3L, chr3R, chr4, chrX and chrY, were included in the analysis. All interchromosomal and intrachromosomal interactions with a distance less than 2 kbp or greater than 10 Mbp from the bait were excluded from the analysis. The dataset contains two bait libraries targeting primarily enhancers and promoters with 26 baits in common to access capture efficiency and reproducibility. For the regions captured by these 26 common baits, only the data from the promoter library were used in the downstream analyses, so as not to duplicate their interactions. The data for these 26 regions in the enhancer library were only used for QC.

To reduce the impact of global differences in P(s) curves at different stages of embryogenesis, we plotted a P(s) curve for each replicate of the five conditions (Extended Data Fig. 1e)—for each E/P bait, the frequency of all observed interactions was calculated. All interactions, across all baits, were then divided into 1 of 30 bins based on the interaction distance. The bins were of equal width, in log space, and the middle of the first and last bins were 2 kbp and 10 Mbp, respectively. The mean interaction frequency of each bin was calculated, and a decreasing monotonic spline was used to fit the log of mean frequencies against the log of bin mid-point using the mgcv package in R (version 1.8-22). The distance-probability fits were subsequently used to correct for global differences in P(s) curves between conditions and replicates by supplying normalization factors to the estimateSizeFactors function in DESeq2 (ref. ^67^; see below). To calculate a normalization factor for an interaction, the expected frequencies of the interaction are calculated based on the interaction distance. Normalization values for the interaction are then calculated by dividing the individual expected frequencies by the geometric mean of all expected frequencies.

To identify differential and constant interactions (Fig. 2b), two separate DESeq2 (ref. ^67^; version 1.16.1) analyses were run, with two distinct null hypotheses, to identify both statistically significant differential interactions and statistically significant constant interactions. For each E/P bait, the intrachromosomal interaction counts across all replicates were combined into a matrix and a DESeq dataset was generated using the samples as the sole design variable. A distance-probability normalization matrix was generated (as described above) and added to the object, and the differential analysis was performed. Differential interactions were identified by testing a null hypothesis that the log_2_(FC) was equal to 0 (|log_2_(FC)| = 0) by supplying the following arguments to the ‘results’ function in DESeq2: lfcThreshold = 0, altHypothesis = greaterAbs. To identify constant interactions, we used an alternative null hypothesis that the absolute log_2_(FC) is greater than one (|log_2_(FC)| ≥1) by supplying the following arguments to the ‘results’ function of DESeq2: lfcThreshold = 1, altHypothesis = lessAbs. Independent filtering was applied in the identification of both differential and constant interactions with a significance cutoff of 0.05 (*α* = 0.05) and the maximum CHiCAGO score across samples was applied as the filter argument. Significance was ascribed to an adjusted *P* value of <0.05 (FDR) with a further requirement for differential and constant interactions to have an absolute nonshrunken log_2_(FC) of >0.7 and <0.4, respectively. In this filtering process, putative differential interactions with an absolute log_2_(FC) of less than or equal to 0.7 (|log_2_(FC)| ≤0.7) were discarded along with putative constant interactions with an absolute log_2_(FC) of more than or equal to 0.4 (|log_2_(FC)| ≥0.4).

Differential interactions were therefore defined as those with <0.05 FDR and >0.7 log_2_(FC), and a significant CHiCAGO score (≥5.0) in one or more conditions to take the distance decay from the bait into account. The identification of differential interactions was highly reproducible, as seen from the 26 common baits (Supplementary Fig. 1h). Constant interactions were defined as those with <0.05 FDR and >0.7 log_2_(FC), against the null hypothesis that the absolute interaction log_2_(FC) is greater than one (|log_2_(FC)| ≥1). The lfcShrink command was applied to generate shrunken log_2_(FC) estimates by using the ‘normal’ shrinkage estimator and was used as the plotted measure of log_2_(FC) in this study.

### Clustering Capture-C data

Clustering of Capture-C replicates across all samples was performed to determine data reproducibility (Supplementary Fig. 2). Raw interaction counts for the 26 baits common to both libraries were extracted and filtered to remove interactions not observed in all replicates. A variance stabilizing transformation (using DESeq2) was applied to the counts of the remaining interactions. Pearson’s correlation coefficient (*r)* was calculated for the transformed counts, and the distance between replicates was calculated using the formula $2\sqrt{\left(1-r\right)}$ (Supplementary Fig. 2).

Hierarchical clustering of the high-confidence interactions only called in one time point (Fig. 1d (colored bars in the UpSet plot)) and the differential interactions called by DESeq2 (Fig. 2b) were performed on the distances using the complete-linkage method to determine how the signal changes over developmental time and tissues (Extended Data Fig. 1a and Fig. 2d). Normalized interaction counts, for all relevant interactions in any comparison, were extracted from the ‘mu’ assay of the DESeqDataSet objects. These counts were log_2_ transformed and then, for every individual interaction, the mean value for all samples was subtracted. Pearson’s correlation coefficient (*r)* was calculated for the transformed counts, and the distance between interactions was calculated using the formula $2\sqrt{\left(1-r\right)}$. Hierarchical clustering was performed using the complete-linkage method. The values in the dendrogram (Fig. 2c) are the recomputed distances upon application of the hierarchical clustering.

To cluster interactions (Extended Data Fig. 1a and Fig. 2d), we performed *k*-mean clustering on the interaction distances described in the previous paragraph. A value of *k* = 6 was chosen for Fig. 2d as this was the highest value that generated clusters with distinct profiles across the samples.

### Insulator, DHS and H3K27ac data analysis

ChIP-seq (BEAF-32, CTCF, Su(Hw) and H3K27ac) FASTQ files were analyzed using standard methods, as described previously^62,63^. Of the 32 ChIP–seq datasets (4 conditions × 4 factors × 2 replicates), 3 replicates (neuro 6–8 h Rep1 Su(Hw); neuro 6–8 h Rep1 CTCF; neuro 10–12 h Rep1 Su(Hw)) failed QC analyses due to low read count and poor enrichment and were excluded. Pseudoreplicates were made from the remaining good replicates for these three conditions, all others used biological replicates. The known motif for BEAF-32, CTCF and Su(Hw) was enriched under the respective ChIP peaks. After peak calling, we generated a consensus set of 10,052 insulator-bound regions, by merging peaks from all experiments whose summit was within 50 bp. Differential peaks were identified using DESeq2 (ref. ^67^). Significance was ascribed to an adjusted *P* value of <0.05, and differential and constant peaks were defined as having both a nonshrunken log_2_(FC) of >0.7 and <0.4, respectively.

### DHS and ChIP enrichment analysis

When integrating Capture-C with ChIP and DHS peaks (Figs. 3 and 4), the following parameters were used: proximity between an E/P bait/otherEnd and ChIP/DHS peak was defined as the two regions being within 500 bp of each other, regardless of orientation. The interaction interval was defined as the region spanning both the bait and otherEnd ±5 kb. For a peak to overlap the interaction interval, there must be an overlap of at least one base.

To identify DHS that are distal to transcriptional start sites (TSSs), release 13 of the Dm6 genome (dmel_r6.13) was used. TSSs are defined as the start of the most 5′ exon for each gene. DHS less than 500 bp from a TSS was defined as TSS proximal and the rest as TSS distal. Fisher’s exact test was used to determine the significance of contingency tables classifying genomic regions by their proximity to a genomic feature. Mann–Whitney tests were used to determine the significance of the frequency at which genomic features were found in genomic intervals compared to a control set of regions.

### TF motif enrichment

*D. melanogaster* motifs from the CIS-BP database^68^ (build version 2) were used for enrichment analysis within test and control sets of DHS (that are tissue/stage matched) and in proximity (<500 bp) to the test and control Capture-C interacting 'other end' regions (loop anchors), respectively. DHS present in both the test and control sets were removed from the control set and kept in the test set. Enrichment of motifs in the test DHS, relative to the control DHS in the same developmental condition (cell type/time point), was performed using AME software^69^ with an adjusted *P* value threshold of 1 × 10^−4^.

### Statistics and reproducibility section

Two independent biological replicates (from the embryo collections on) were used for all experiments (Capture-C, ChIP–seq with insulators and H3K27ac). QC analyses were used to assess reproducibility between replicates. No statistical methods were used to predetermine sample sizes, but our sample sizes are similar to those reported in previous publications using the same methods^11,14,43^. The statistical tests used in this study (Fisher’s Exact test and Wilcoxon test) are nonparametric and therefore do not make assumptions about a normal distribution. The experiments were not randomized. Data collection and analysis were not performed blind to the conditions of the experiments. The embryo images shown in Fig. 5 and Extended Data Figs. 4 and 5 are representative images from at least five embryos showing similar expression at that stage.

### Reporting summary

Further information on research design is available in the Nature Portfolio Reporting Summary linked to this article.

## Online content

Any methods, additional references, Nature Portfolio reporting summaries, source data, extended data, supplementary information, acknowledgements, peer review information; details of author contributions and competing interests; and statements of data and code availability are available at 10.1038/s41588-024-01678-x.

### Supplementary information

Supplementary Information Supplementary Figs. 1–5, Supplementary Note and Supplementary Methods.

Reporting Summary

Supplementary Table 1 QC metrics from HICUP, annotated bait activity, activity of tested enhancer, additional mapping information, cloning enhancer assays, BAC probes, blocking oligos, adapter sequences, antibodies, ESTs used for in situs.

Supplementary Data 1 Capture-C interaction data—all and differential.

Supplementary Data 2 DHS data—tissue and stage matched.

Supplementary Data 3 H3K27ac ChIP–seq data—tissue and stage matched.

Supplementary Data 4 BEAF-32 ChIP–seq data—tissue and stage matched.

Supplementary Data 5 CTCF ChIP–seq data—tissue and stage matched.

Supplementary Data 6 Su(Hw) ChIP–seq data—tissue and stage matched.

## Data Availability

All raw data were submitted to EMBL-EBI’s ArrayExpress under accessions: E-MTAB-9310 (Capture-C data) and E-MTAB-12639 (ChIP–seq data). https://www.ebi.ac.uk/biostudies/arrayexpress/studies/E-MTAB-9310?key=9abe1e3e-f26e-4a6d-84cb-0ef5b3fa555dhttps://www.ebi.ac.uk/biostudies/arrayexpress/studies/E-MTAB-12639?key=9fad869f-e656-475c-aa76-0dfdf06be384 The processed data are available in Supplementary Data 1–6. We also generated a user-friendly searchable shiny app, which has all Capture-C interaction maps, and tissue-specific insulator and H3K27ac ChIP–seq peaks, where one can visualize the data for all ~600 E–P baits: http://furlonglab.embl.de/data/E-P_CaptureC.

## Code availability

There was no custom software used in this study. All analyses were performed with standard published software, as described in the Methods.
