# Supplementary material for: Enhancer–promoter interactions become more instructive in the transition from cell-fate specification to tissue differentiation
Source: Nat Genet. 2024 Mar 11;56(4):686–96. doi: 10.1038/s41588-024-01678-x (PMC11018526; doi:10.1038/s41588-024-01678-x)
Supplement: Supplementary file 1 — Supplementary Figs. 1–5, Supplementary Note and Supplementary Methods. [file 41588_2024_1678_MOESM1_ESM.pdf]

nature genetics

Article

https://doi.org/10.1038/s41588-024-01678-x

Enhancer–promoter interactions become more instructive in the transition from cell-fate specification to tissue differentiation

In the format provided by the authors and unedited

INVENTORY OF SUPPLEMENTARY INFORMATION

Supplementary Fig. 1: Overview of the experimental design and data quality

Supplementary Fig. 2: Capture C is highly reproducible across replicates and experiments

Supplementary Fig. 3: Differential interactions in relation to activity and CHiCAGO score

Supplementary Fig. 4: The relationship between insulator binding and E/P interactions

Supplementary Fig. 5: Differential interactions involving non-coding RNAs

Supplementary Note:

Capture-C in the early blastoderm (2-3h)

Comparison of 2-3h whole embryo E/P interactions with later time-points

Drosophila insulator proteins

Supplementary Methods

Isolation of nuclei for Capture-C and ChIP-seq using FANS (detailed protocol)

Capture-C in specific tissues and stages (detailed protocol)

Tissue-specific ChIP-seq on insulator proteins and H3K27ac

DNA and RNA fluorescence in situ hybridization (FISH) in *Drosophila* embryos

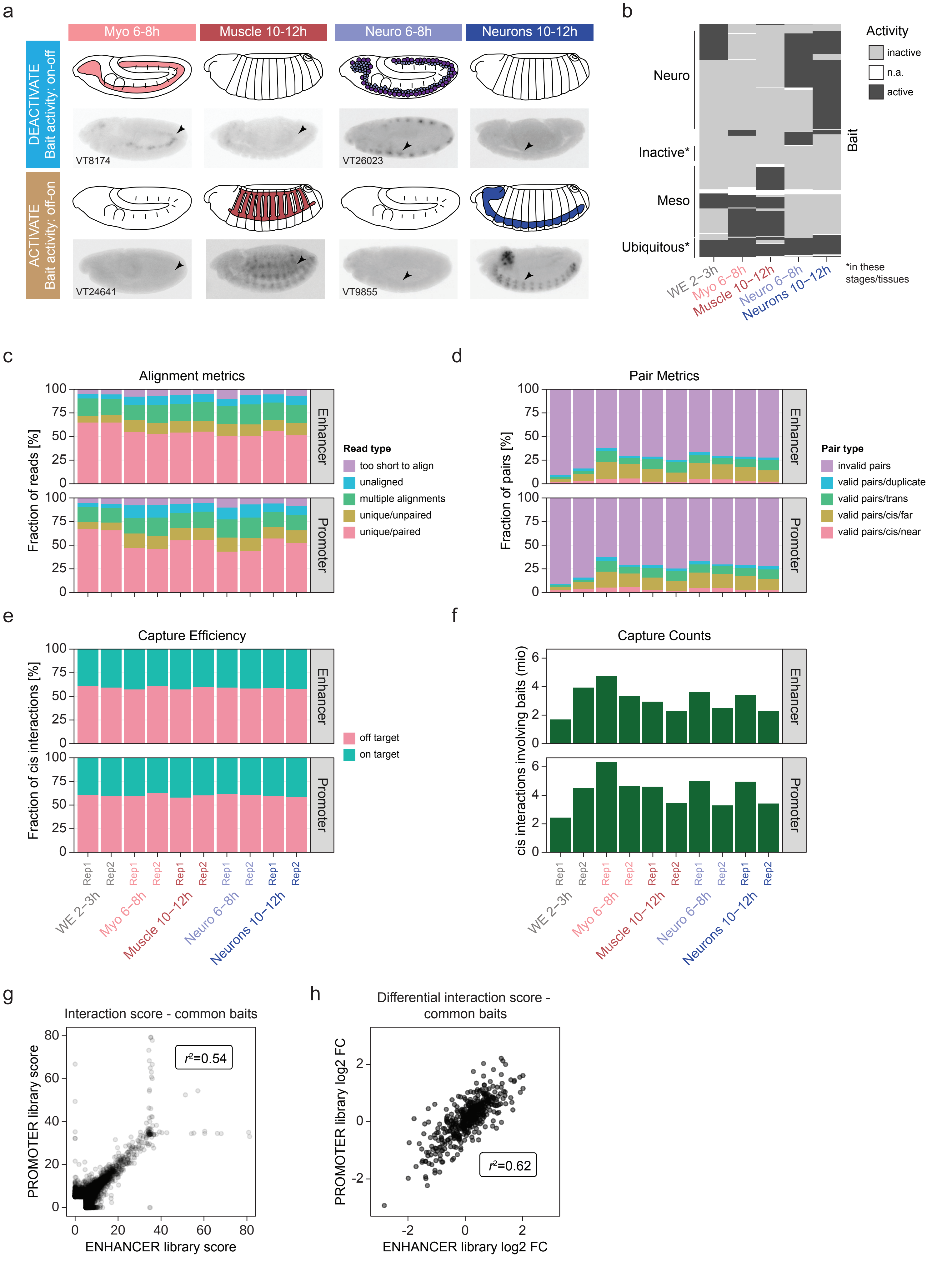

a
Myo 6-8h
Muscle 10-12h
Neuro 6-8h
Neurons 10-12h
DEACTIVATE
Bait activity: on-off
ACTIVATE
Bait activity: off-on
VT8174
VT26023
VT24641
VT9855
b
Neuro
Inactive*
Meso
Ubiquitous*
Bait
Activity
inactive
n.a.
active
WE 2–3h
Myo 6–8h
Muscle 10–12h
Neuro 6–8h
Neurons 10–12h
*in these stages/tissues
c
Alignment metrics
Fraction of reads [%]
Enhancer
Promoter
Read type
too short to align
unaligned
multiple alignments
unique/unpaired
unique/paired
d
Pair Metrics
Fraction of pairs [%]
Pair type
invalid pairs
valid pairs/duplicate
valid pairs/trans
valid pairs/cis/far
valid pairs/cis/near
e
Capture Efficiency
Fraction of cis interactions [%]
off target
on target
f
Capture Counts
cis interactions involving baits (mio)
Rep1
Rep2
g
Interaction score - common baits
PROMOTER library score
ENHANCER library score
r²=0.54
h
Differential interaction score - common baits
PROMOTER library log2 FC
ENHANCER library log2 FC
r²=0.62

Supplementary Figure 1: Overview of the experimental design and data quality

(a) Examples of developmental enhancers with dynamic tissue-specific activity, which were used as baits. *Top row*: Schematic and embryo images of enhancers active in Myoblasts (Myo) or Neuronal (Neuro) cells at 6-8h, but not at 10-12h (ON-OFF). Images show the reporter genes' expression driven by the enhancer element. *Bottom row*: Schematic and embryo images of enhancers not active in Myo or Neuro at 6-8h, but are active in Muscle or Neurons at 10-12hr (OFF-ON). Embryo images show *in situ* hybridizations of the transgenic reporter, obtained from the Fly Enhancer VDRC database (https://enhancers.starklab.org/). The vast majority of enhancers and promoters were selected based on their exclusive activity in the developing mesoderm/muscle and nervous system matching one of the categories ON-ON, OFF-OFF, OFF-ON, ON-OFF. Arrowheads indicate reporter signal localization (enhancer activity) in the 'ON' state, in the 'OFF' state there is no signal detected. **(b)** Heatmap indicating the binary activity of all selected baits. Data is discretized to show active (dark grey), inactive (light grey) or unknown (n.a.) based on signal in the respective databases (e.g. VDRC, CAD4, BDGP). The respective tissue is indicated underneath. Enhancers/promoters that are dynamic in their activity between 6-8h and 10-12h (tissue specification and terminal differentiation) and tissue-specific were selected. Baits were included for ubiquitously expressed or non-expressed genes as controls. Tissue-/stage-specific analysis was performed using only the baits with known annotated activity in both conditions. **(c-f)** Capture-C quality control assessment from HiCUP: **(c)** Stacked bar chart; displaying alignment metrics classifying individual reads in the alignment portion of HiCUP violet = too short to align after trimming to the first occurrence of the DpnII motif, cyan = unaligned/not mapped to the genome, green = aligned to multiple genomic regions, brown = mapped to a single location with not mapped mate, pink = mapped reads to a single location with a uniquely mapped paired end mate. **(d)** Stacked bar chart depicting pair metrics for "unique alignment paired" reads from (c); violet = invalid pairs, cyan = duplicate valid read pairs, green = valid read pairs with *trans* ligation events, brown = valid pairs in *cis* with fragments separated by more than 10 kb, pink = valid pairs in *cis* with fragments separated by less than 10 kb. **(e)** Stacked bar chart depicting capture efficiency for valid read pairs in *cis* from (d); pink = read pairs not containing bait sequence, turquoise = read pairs with bait sequence. **(f)** Bar chart depicting the number of capture counts (*cis* interactions) for all baits. Displayed is the number of interactions for "on target" events from (e). The proportion of valid, aligned and paired-end read pairs (panels c,d) is very similar across conditions, except the 2-3h time-points which is lower. The Capture-C efficiency (panel e) and number of Capture counts (panel f) is comparable across all conditions, including 2-3h. **(g)** To assess reproducibility, we compared CHiCAGO scores generated from the 26 baits present in both the Enhancer and Promoter libraries (which were each done with biological replicates). Overall, there is a very strong positive correlation between the scores generated in each library (r^2=0.54) with clear diversion occurring only for interactions with extremely significant CHiCAGO scores (>30) in both libraries. **(h)** To assess reproducibility of the calculated log2 fold-change, we compared log2 fold-change values generated for the 26 baits present in both the Enhancer and Promoter libraries. There is a clear and strong correlation (r^2=0.62) between the values generated in each library showing the reproducibility of both the experimental data and its analysis, including the identification of differential interactions.

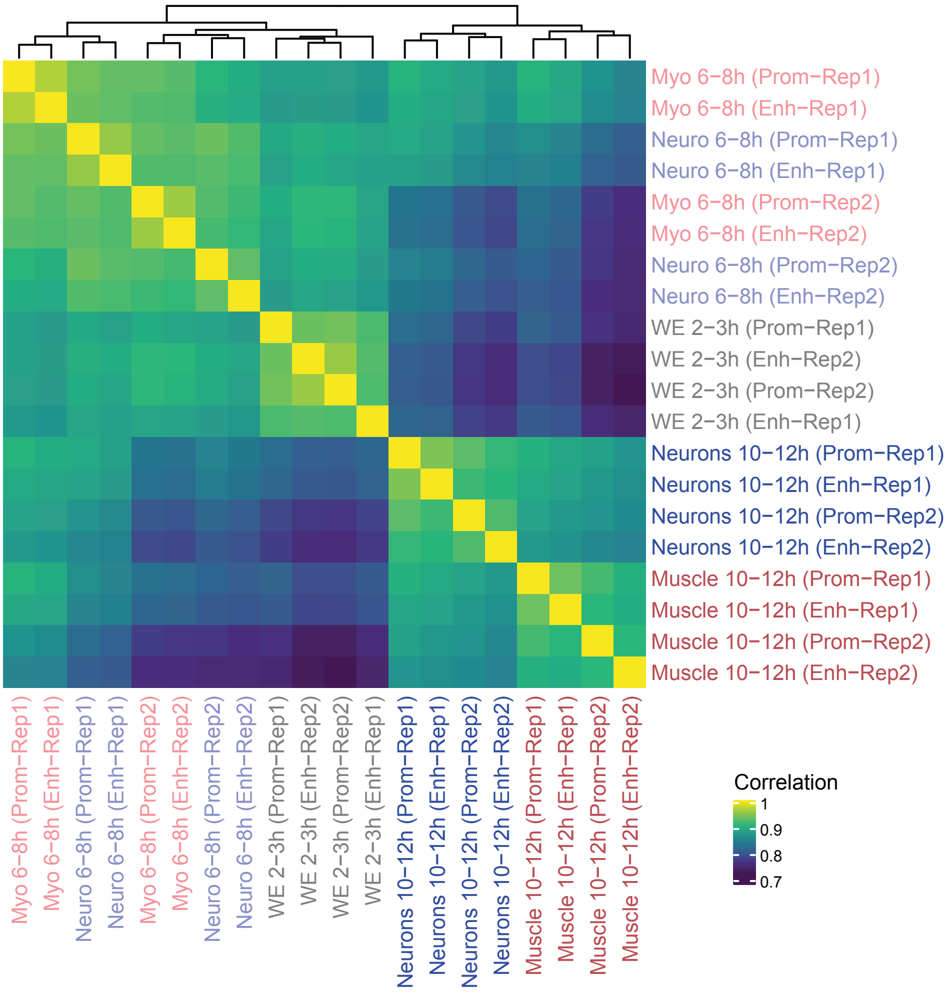

Supplementary Figure 2: Capture-C is highly reproducible across replicates and experiments Heatmap showing correlation of Capture-C interaction counts between replicates, samples, and enhancer/promoter bait libraries for each Capture-C experiment. Correlation is calculated for the 26 baits common to both bait libraries. The first level of clustering are the libraries generated from the same replicate. The data subsequently clusters by sample and then timepoint. Overall, there is very good correlation between data generated from the same sample and condition.

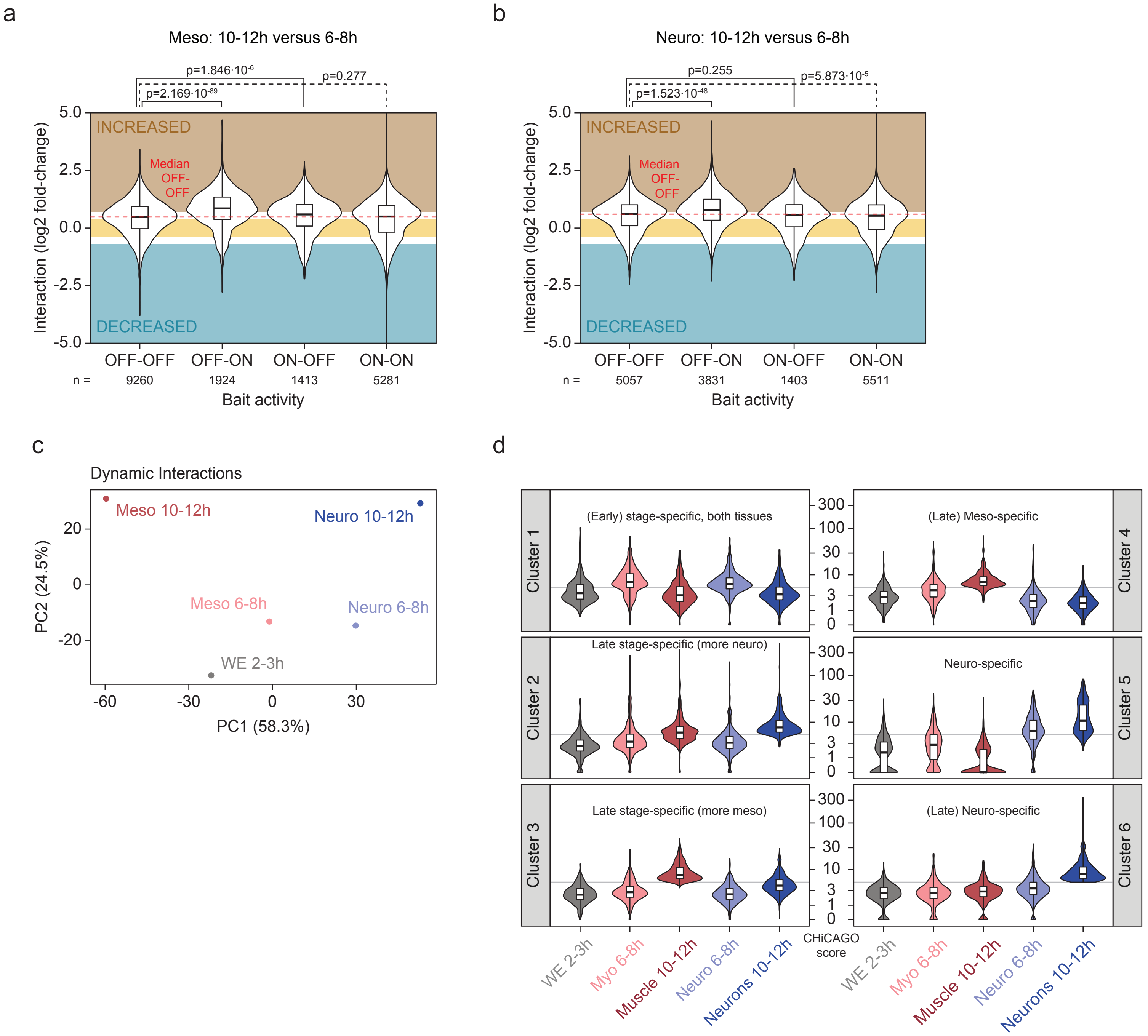
a
Meso: 10-12h versus 6-8h
p=1.846·10-6
p=0.277
p=2.169·10-89
INCREASED
Median OFF-OFF
DECREASED
Interaction (log2 fold-change)
OFF-OFF
OFF-ON
ON-OFF
ON-ON
n =
9260
1924
1413
5281
Bait activity
b
Neuro: 10-12h versus 6-8h
p=0.255
p=5.873·10-5
p=1.523·10-48
INCREASED
Median OFF-OFF
DECREASED
Interaction (log2 fold-change)
OFF-OFF
OFF-ON
ON-OFF
ON-ON
n =
5057
3831
1403
5511
Bait activity
c
Dynamic Interactions
Meso 10-12h
Neuro 10-12h
Meso 6-8h
Neuro 6-8h
WE 2-3h
PC2 (24.5%)
PC1 (58.3%)
d
Cluster 1
(Early) stage-specific, both tissues
(Late) Meso-specific
Cluster 4
Cluster 2
Late stage-specific (more neuro)
Neuro-specific
Cluster 5
Cluster 3
Late stage-specific (more meso)
(Late) Neuro-specific
Cluster 6
CHiCAGO score
WE 2-3h
Myo 6-8h
Muscle 10-12h
Neuro 6-8h
Neurons 10-12h

Supplementary Figure 3: Differential interactions in relation to activity and CHiCAGO scores

(a) Violin plots/boxplots showing changes in interaction frequencies (log2-fold) of all significant interacting regions for enhancers/promoters baits changing in their activity between 6-8h and 10-12h (OFF-OFF, OFF-ON, ON-OFF, ON-ON) in the muscle lineage. Number (n) of interacting fragments (CHiCAGO score ≥5) indicated underneath. p-value (above) from non-parametric Wilcoxon test (two-sided) shows a significant concordant trend for increased interaction frequencies comparing baits going from OFF-OFF to OFF-ON. **(b)** Similar to (a) comparing changes in interaction frequencies (log2-fold) in the nervous system from 6-8h to 10-12h, for E/P baits going from (OFF-OFF, OFF-ON, ON-OFF, ON-ON). p-value (above) from non-parametric Wilcoxon test (two-sided). Comparing E/Ps that go from OFF-OFF to OFF-ON, there is a concordant increase in the median interaction signal. A concordant decrease in interaction signal form E/Ps that go from ON-OFF is not observed in both the muscle (a) and neuronal (b) lineages – most likely due to the smaller number of enhancers/conditions in these comparisons compared to Fig. 2a. We note there is also a small increase in ON-ON neuronal E/Ps, compared to the OFF-OFF condition, suggesting that a subset of active E/P baits increase their interaction frequency (either strengthened or new interactions) between these two stages, which may reflect an increase in the gene or enhancer activity in the second time-point (which is not reflected in the binary 'ON' annotation). **(c)** Principle Component Analysis (PCA) of all differential interactions (from Fig. 2d) in at least one tissue/time comparison (same interactions used for the dendrogram in Fig. 2c). **(d)** Violin plots/box plots depicting the CHiCAGO scores of all interactions within a given cluster (Fig. 2d) in the 5 tested conditions. The applied CHiCAGO score threshold (≥5) is indicated by the horizontal grey line. The plot highlights that the majority of interactions in a cluster (Fig. 2d) are only significant (score ≥5) in the appropriate condition(s), confirming their strong enrichment in time- and/or tissue-specific interactions. In cluster 5, for example, the majority of differential interactions are only significant (score ≥5) in the nervous system, and have an even higher score at 10-12h. Cluster 6, the majority of interactions are exclusive to the nervous system at 10-12h (stage of differentiation). For boxplots in a, b, d: centre=median, upper and lower bounds=interquartile range, whiskers=minimum and maximum)

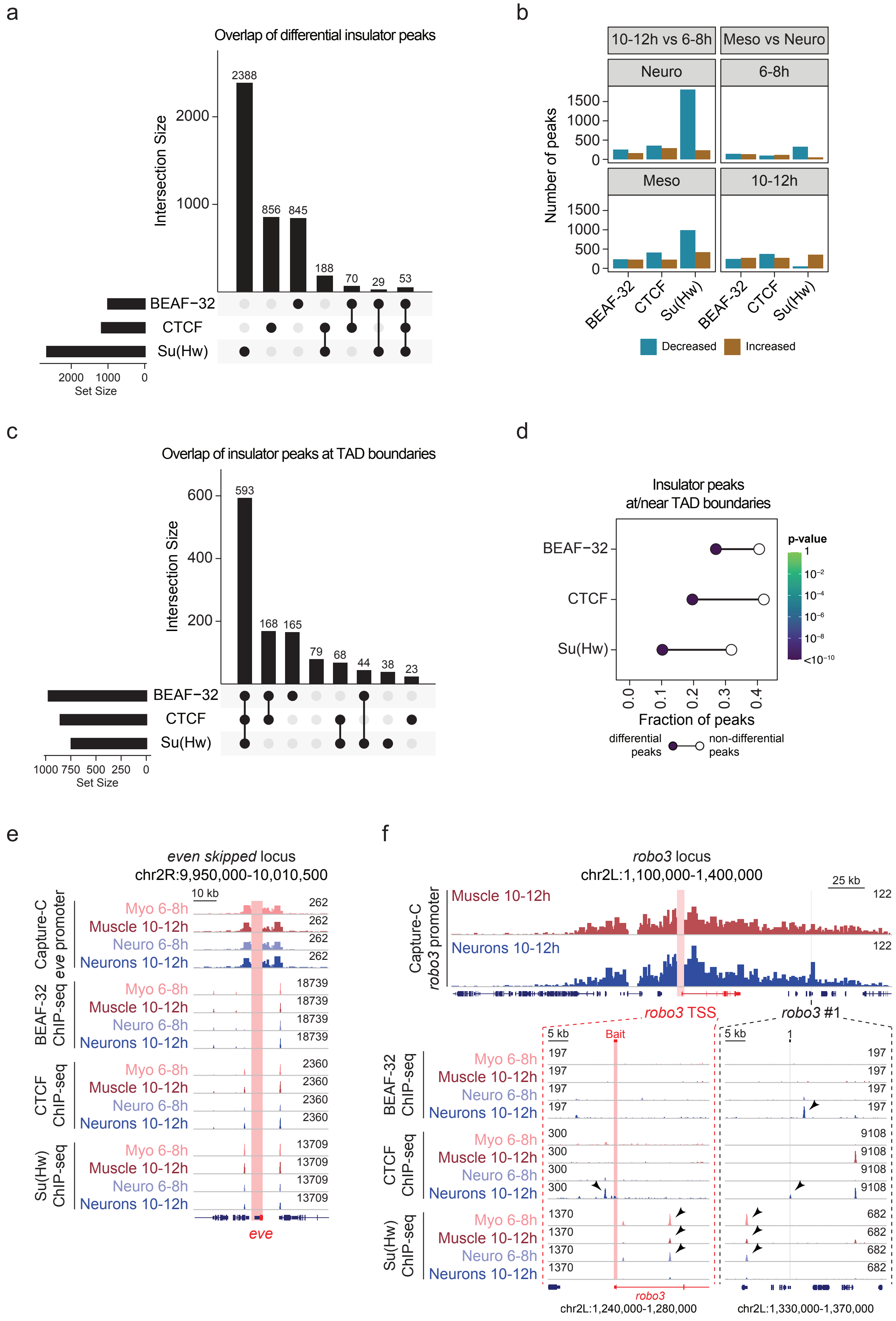

a
Overlap of differential insulator peaks
Intersection Size
2388
856
845
188
70
29
53
BEAF−32
CTCF
Su(Hw)
Set Size
b
10-12h vs 6-8h
Meso vs Neuro
Neuro
6-8h
Meso
10-12h
Number of peaks
Decreased
Increased
c
Overlap of insulator peaks at TAD boundaries
Intersection Size
593
168
165
79
68
44
38
23
d
Insulator peaks at/near TAD boundaries
p-value
Fraction of peaks
differential peaks
non-differential peaks
e
even skipped locus
chr2R:9,950,000-10,010,500
Capture-C eve promoter
BEAF-32 ChIP-seq
CTCF ChIP-seq
Su(Hw) ChIP-seq
Myo 6-8h
Muscle 10-12h
Neuro 6-8h
Neurons 10-12h
eve
f
robo3 locus
chr2L:1,100,000-1,400,000
Capture-C robo3 promoter
robo3 TSS
robo3 #1
Bait
robo3
chr2L:1,240,000-1,280,000
chr2L:1,330,000-1,370,000

Supplementary Figure 4: The relationship between insulator binding and E/P interactions

(a) UpSet plot showing the overlap of differential insulator peaks. For each insulator protein the differential peaks across all samples were merged prior to determining their overlaps. **(b)** Bar chart depicting the number of decreased (turquoise) and increased (brown) insulator peaks in the respective conditions based on a differential analysis (DESeq2) of ChIP-seq signal. **(c)** UpSet plot showing the overlap of insulator peaks with TAD boundaries (within 5 kb) as annotated in Sexton et al., 2012. The majority of TAD boundaries are bound by two or all three insulators. **(d)** Enrichment of differential (filled circle) or non-differential insulator peaks at TAD boundaries. Plot shows frequency of insulator peaks within 5kb of the TAD boundary. Color shade of filled circles indicates p-value (Fisher exact test, two-sided). Non-differential insulator peaks are more likely to be found near or at TAD boundaries. **(e)** Normalized Capture-C and insulator ChIP-seq signal in the indicated conditions at the *even skipped* locus, bait (*eve* promoter) highlighted by red bar. The small *eve* regulatory landscape is flanked by invariant insulator binding. Interaction frequency decreases substantially left and right outside of insulator binding sites. **(f)** Example of differential tissue-specific interactions with tissue-specific insulator binding at *robo3* locus. Interaction between the *robo3* promoter (bait = red vertical bar) and the *robo3* #1 element in Neuro 10-12h is associated with CTCF binding close to the *robo3* promoter (and BEAF-32 close to the *robo3* #1 enhancer) specifically in Neurons 10-12h (arrows).

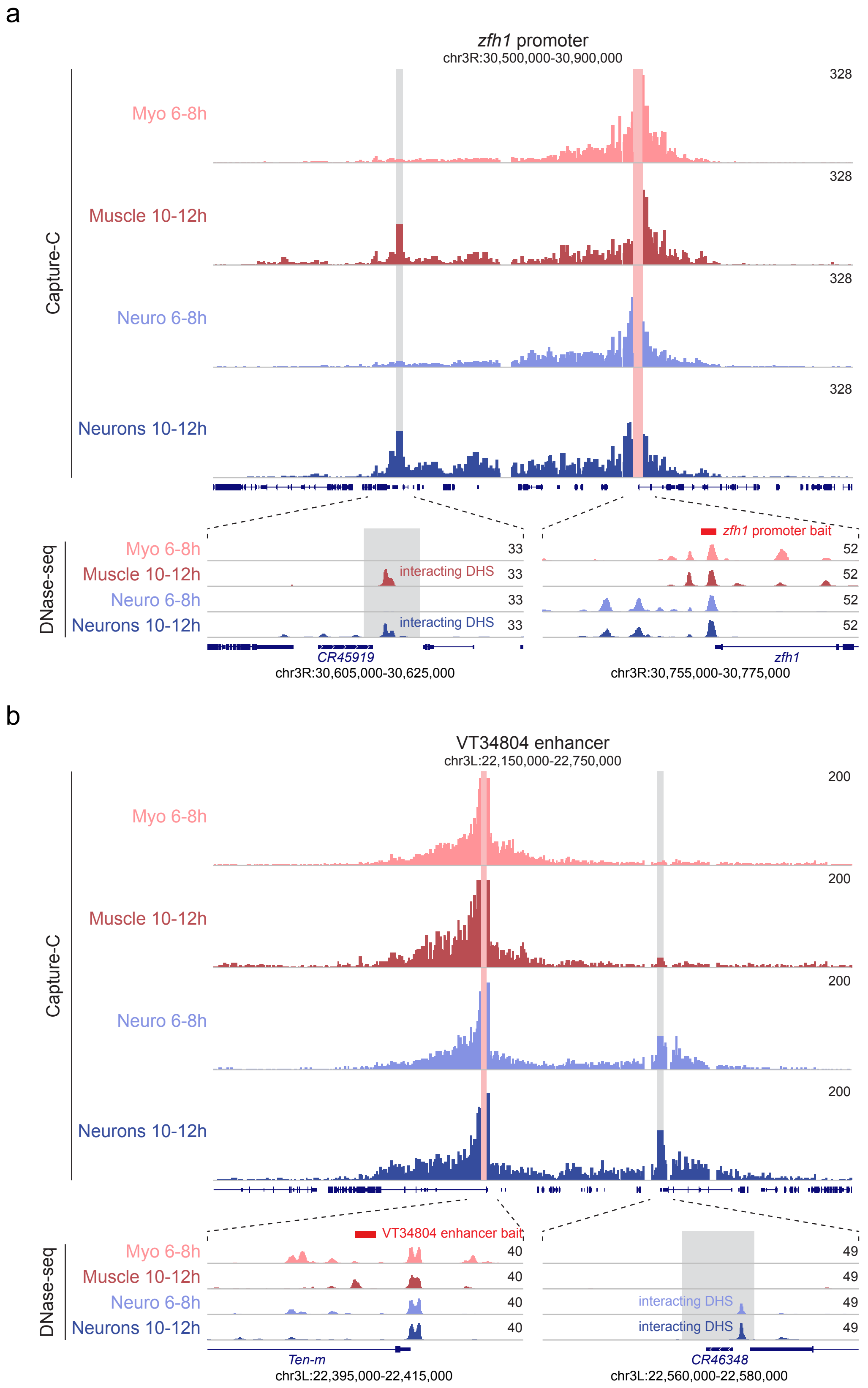

Supplementary Figure 5: Differential interactions involving non-coding RNAs
(a) Normalized Capture-C signal at the zfh1 locus in 4 conditions. Vertical red bar = bait (zfh1 promoter). Below, zoomed in view of DHS signal in the same 4 conditions and gene models at the two ends of the loop. The lncRNA (CR45919) is indicated (left). The zfh1 promoter-CR45919 interaction spans 150 kb. **(b)** Normalized Capture-C signal from the enhancer (VT34804) used as bait (vertical red bar). Below, zoomed in view of DHS signal in the same 4 conditions and gene models at the two ends of the loop. The lncRNA (CR46348) is indicated (right). Enhancer VT34804-CR46348 interaction spans 164 kb. Grey bar = fragment at differential interaction with highest interaction frequency in that region.

SUPPLEMENTARY NOTE

Capture-C in the early blastoderm (2-3h)

To better understand the topological 'ground state' of enhancers and promoters very early in development we included a 2-3h timepoint in our collections. Such collections are always shifted to a smaller timeframe, as it takes some time (15-20min) for the adults to settle down and start laying embryos again. So effectively a collection at 2-3h (after egg laying) is a ~2-2:40 min collection, mainly spanning nuclear cycles 13 and 14 (very early stage 5) of the early blastoderm stage prior to, and around the time of, the mid-blastula transition and zygotic genome activation. At this stage the very rapid syncytial cell divisions are being completed and TADs are just starting to be established. We, and others, showed by microscopy that there is still extensive cell-to-cell heterogeneity in TAD formation at this time-window[1,2], and the insulation strength of the newly formed boundaries is lower[2,3].

Perhaps because inter-TAD insulation and chromatin topology have not been fully established across all cells at this stage, we noted certain potentially interesting differences from the other time-points, that were outside the scope of this study. For example, looking at the relationship between E/P interactions and TADs, the majority do not cross a TAD boundary, as expected, with the exception of some very long-range loops at the megabase scale, as also seen in other studies[4-7] (Extended Data Fig. 1f). However, the very early 2-3h sample has the lowest percentage of (i) intra-TAD high-confidence interactions (Extended Data Fig. 1g) and (ii) interactions that do not cross any TAD boundaries (Extended Data Fig. 1f). Conversely, at 2-3h more interactions cross 4 or more TAD boundaries compared to the other time-point (Extended Data Fig. 1f). While we cannot rule out that this is technical[*], it might reflect the lower insulation between TADs at 2-3h as they are still not established in many cells at this stage of embryogenesis[1,2]. This could also account for the longer distance of 2-3h interactions compared to 6-8h (Extended Data Fig. 1d), many of which have very low read counts.

[*]*We note that the proportion of valid, aligned and paired-end read pairs for 2-3h is a bit lower than at the other time points (Supplementary Fig. 1d, Supplementary Table), however the Capture Efficiency (Supplementary Fig. 1e) and Capture counts combining both replicates (Supplementary Fig. 1f) at 2-3h is comparable to the other conditions. The overlap of 2-3h interactions defined by CHiCAGO as significant (score >5) to stage matched DHS is lower compared to the other time-points. This may reflect a lower quality of the Capture-C data, although there are no indications in QC metrics that could account for this (see above), or alternatively the lower number of DHS peaks in the Reddington et al dataset[8] at the overlapping 2-4h time-window – which contains 7,423 DHS at 2-4h, while the other 4 conditions have more than 18,000 peaks (Figure 1b in Reddington et al.[8]).*

Comparison of 2-3h whole embryo E/P interactions with later time-points

E/P interactions were assessed by two means in this study:

a) To examine all E/P interactions, a high-confidence set was defined as interactions with (i) a CHiCAGO score >5 and (ii) overlapping a DHS at that stage/tissue (data from Reddington et al.[8])

b) Differential interactions, defined as being significantly differential by DESeq2 between pair-wise comparisons, using (i) < 0.05 (FDR), (ii) log2 fold-change of > 0.7, and (iii) post-

filtering for interactions with a CHiCAGO score > 5.0, to take distance from the bait into account.

Many interactions called significant by CHiCAGO (score >5) at 2-3h were not significant at 6-8h, or significantly differential (or constant) between 6-8h and 2-3h. These were generally very low frequency, long-range interactions, which may represent spurious, rather low frequency interactions due to a lack of insulation at that particular stage. Many of these do not overlap DHS at 2-3h, and were therefore excluded from our set of high-confidence interactions (described in Fig. 1). Nevertheless, we note that the high-confidence interactions at 2-3h are also on average longer distance interactions compared to 6-8h (Extended Data Fig. 1d), and many are not called as significant in 6-8h samples. Consequently, although the number of high-confidence interactions is roughly the same between 2-3h and 6-8h, in both myoblasts and the nervous system (Fig. 1c), there is low overlap between the first two stages (2-3h and 6-8h, Fig 1d, UpSet plot). However, the majority of these interactions are not significantly differential (defined by DESeq2) between 2-3h and 6-8h. Actually, the number of significantly constant interactions is higher than the number of differential interactions between 2-3h and 6-8h (Fig. 2b).

This is much more dramatic just one hour later in development, as seen in our previous 4C study of 100 enhancers comparing 3-4h (stages 6-7, post-gastrulation embryos) to 6-8h[9]. During this additional hour, the vast majority of E-P interactions between the two time-points are constant (with only ~6% differential), suggesting that the 6-8h interactions were already pre-formed at 3-4h. The timing (i.e. the difference between 2-3h and 3-4h) coincides with the transition between the initial early stages of TAD formation, to when TADs are well established with much lower cell-to-cell heterogeneity and stronger insulation[1-3]. Although correlative, this suggests that in early blastoderm embryos (at 2-3 hours), enhancers and promoters may contact other regions (often at low frequency), and that this is 'locked down' later in embryogenesis, perhaps after TADs are firmly established. Alternatively, it may reflect some other property of chromatin organisation (high motility or flexibility) at very early stages of embryogenesis.

Drosophila insulator proteins

Drosophila have many insulator proteins. Here, we focused on three major ones, CTCF, BEAF-32 and Su(Hw), that bind to the majority of domain boundaries[2,4,10,11] and have been implicated in gene regulation[12,13]. *Drosophila* CTCF is not required for embryogenesis[14], but is required for the expression of specific genes [14,15]. Similarly, BEAF-32 depletion results in mis-regulation of some genes[12,16]. Su(Hw) binds to gypsy retrotransposons, which acts as an insulator to block E-P communication[17].

SUPPLEMENTARY METHODS

Isolation of nuclei for Capture-C and ChIP-seq using FANS (detailed protocol)

Prior to sorting, ~200 million dissociated nuclei/ml were incubated with primary antibody over night at 4°C on a nutator, as follows: monoclonal mouse anti-Elav to mark neuronal cells and rabbit anti-Mef2 to mark all mesoderm and muscle derivatives (Supplementary Table). Following primary antibody incubation, nuclei were washed 3x in 10 ml PBTB-NP-40 and resuspended in PBTB (1xPBS, 5% BSA, 0.1% Triton-X-100, supplemented with Complete Protease Inhibitor, Roche). Secondary antibodies (listed in Supplementary Table) were incubated for 1h at 4°C in PBTB, followed by three washes in 10 ml PBTB, and then resuspended, counterstained with DAPI and subjected to FANS. FANS was performed using MoFlo Cell Sorters (Beckman Coulter) using our optimized protocol as previously described[8,18,19], isolating nuclei for the myogenic (Mef2+) and neurogenic (Elav+) lineages. We also actively sorted for non-meso and non-neuro (NM/NN) nuclei (Mef2-/Elav-) representing a mixture of ectoderm and endodermal tissues at both 6-8h and 10-12h, and include this set of 12 Capture-C datasets (4 NM/NN 6-8h, 8 NM/NN 10-12h) as a resource in supplementary material. Only collection tubes with >95 % purity for the gated population were used (most exhibited >98 % purity). Sorted nuclei were pelleted by centrifugation at 3,200xg in a swing-out rotor for 15 min at 4°C and transferred in a small amount of PBT to 1.5-mL LoBind tubes and pelleted again at 3,200xg in a tabletop centrifuge for 15 min at 4°C. The nuclear pellet was snap frozen at -80°C for later use in Capture-C or ChIP-seq experiments.

Capture-C in specific tissues and stages (detailed protocol)

To provide a high-resolution view of E-P interactions, a 4bp cutter (DpnII) was used for the Capture-C, providing a theoretical resolution of ~254bp, and all libraries were sequenced to a high sequencing depth. To ensure enough biological complexity to capture interactions for all regulatory elements, 100 million sorted snap frozen nuclei were used per replicate (per condition), which were resuspended in 1 mL ice-cold permeabilization buffer (10 mM Tris-HCl pH 8.0, 10 mM NaCl, 0.2% (v/v) NP-40 supplemented with Complete Protease Inhibitors without EDTA). Nuclei were handled in aliquots of 25 million. For each aliquot, nuclei were gently dislodged and resuspended with a cut P1000 pipet tip. The resuspended nuclei were transferred to 15-ml polypropylene tubes containing 11.5 mL permeabilization solution (in total 4 tubes of 25 million for 100 million nuclei per replicate sample). The total suspension of 25 million nuclei in 12.5 mL permeabilization buffer was incubated on nutator for 30 minutes at 4°C. After incubation, nuclei were pelleted at 600xg at 4°C for 10 minutes. The supernatant except 1 mL were aspirated, and the remaining nuclei suspension was transferred to a new 1.5-ml tube and pelleted at 600xg and 4°C for 10 minutes. The supernatant was aspirated and the nuclei resuspended in 800 µl ice-cold 1.2x DpnII buffer (NEB) and mixed by inversion. Nuclei were pelleted again at 600xg and 4°C for 10 minutes. The supernatant was aspirated and nuclei resuspended in 400 µl 1.2x DpnII buffer. At this step each reaction tube contains 25 million nuclei in 400 µl 1.2x DpnII buffer (with 4 tubes per replicate sample). 6 µl 20% (w/v) SDS was added to each tube to a final concentration of ~0.3%. The samples were incubated for 1 hour at 37°C and 950 rpm in a thermomixer. After incubation, 40 µl 20% (v/v) Triton-X-100 was added to a final concentration of ~1.8% and the samples incubated for 1 hour at 37°C and 950 rpm in a thermomixer. After incubation, two aliquots of 15 µl (750 U) DpnII (NEB, 50,000 U/mL) were added to each sample several hours apart and the samples digested for 16-24 hours at 37°C and 950 rpm in a thermomixer.

The next day, nuclei were pelleted at 600xg at 4°C for 10 minutes. The supernatant was aspirated (as much as possible) and the nuclei resuspended in ligation buffer (66 mM Tris-HCl pH 7.5, 5 mM $MgCl_2$, 5 mM DTT, 1 mM ATP, 100 ng/µl BSA (NEB), 240 U T4 DNA Ligase (ThermoFisher Scientific)). Samples were incubated for ³ 6 hours at 16°C in a thermomixer.

After the ligation, 5 µl proteinase K (20 mg/mL) was added to a final concentration of 100 ng/µL and the samples incubated at 65°C over-night (O/N) in a thermomixer. The next day, samples were allowed to cool to room temperature. 30 µl RNase A (Fermentas, 10 mg/mL) were added and the samples incubated at 37°C for at least 45 minutes in a thermomixer. DNA was isolated using phenol-chloroform extraction, and precipitated in multiple volumes of 400 µl by adding 1 µl glycogen (Fermentas, 20 mg/mL), 1/10 volume 3M sodium acetate pH 5.2 and 2.5 volumes pre-cooled ethanol and incubated O/N at -80°C. Following centrifugation at ³ 16,000xg at 4°C for 30 minutes the pellet was washed twice with 1 mL cold 70% (v/v) ethanol and resuspended in 100 µl 10 mM Tris-HCl pH 7.5 (for each replicate). Digest and re-ligation was confirmed by analysis on an agarose gel, and DNA concentration determined by Qubit BR or HS DNA kit, following the manufacturer's instructions. For fragmentation, up to 6 µg of DNA, in a total volume of 120 µl, was sonicated to ~200bp using a Covaris S2 sonicator (Settings: Intensity 5, Duty cycle 10%, 200 cycles per burst, 6 cycles – each 1 minutes, Frequency sweeping mode). Sonicated samples were transferred to a new 0.5-mL tube and the DNA size selected using SPRIselect beads (1.8x volume) and recovered in approx. 60 µl water.

1 µg sonicated and size-selected DNA were used for library preparation for each sample. End repair, dA tailing and adapter ligation were performed using the NEBNext Ultra DNA Library Prep Kit II, following the manufacturer's instructions, followed by size-selection using 1 volume of SPRIselect beads. DNA was eluted from beads in 35 µl water. Adapter sequences are listed (Supplementary Table - Adapter sequences). PCR amplification was performed using 25 µl NEBNext Q5 Hot Start HiFi PCR master mix and 5 µl PE 1.0 (P5) and 5 µl PE 2.0 (P7) (each to a final concentration of 1 µM), which was added to 15 µl library DNA. PCR cycles was as follows: 98°C for 30 seconds, 6 cycles of 98°C for 10 seconds and 65°C for 75 seconds, 65°C for 5 minutes and hold at 10°C. The reaction was cleaned up using 0.9 volumes of SPRIselect beads following the manufacturer's instructions, and eluted in 30 µl water. Libraries were analyzed using Qubit dsDNA BR Assay kit and Bioanalyzer.

The first Capture was performed using the Nimblegen SeqCap EZ Hybridization and wash kit, following manufacturer's instructions. Multiplexed libraries were mixed at equimolar ratios prior to promoter and enhancer capture and captured together. Each capture was performed using 1 µg DNA of mixed, multiplexed library DNA, which was incubated with 10 µl Nimblegen SeqCap EZ Developer Reagent and 2 µl of blocking oligo pool (1 nmol/µl, Supplementary Table - Blocking oligos). The samples were lyophilized/dried in a vacuum and resuspended in 10.5 µl hybridization solution (7.5 µl 2x hybridization buffer and 3µl hybridization component A) per capture. Resuspended DNA was denatured at 95°C for 10 minutes and added to 4.5 µl pre-warmed biotinylated probe pool (2.9 µM concentration) in a 0.5 ml DNA LoBind tube and incubated at 47°C for 72 hours. Streptavidin magnetic beads (Dynabeads M-270) were prepared according to the manufacturer's instructions, and the 15 µl hybridization mix added to the magnetic beads and incubated at 47°C. Beads with captured DNA were washed as follows: rinse with 100 µl wash buffer I (47°C), 2x 5 minutes with 200 µl stringent wash buffer (47°C), 2 minutes with wash buffer I (RT), 1 minute with wash buffer II (RT 30 seconds with wash buffer III (RT). Washed beads were resuspended to a total volume of 40 µl.

PCR amplification of captured DNA was performed using KAPA HiFi HotStart ReadyMix. 25 µl of mix was added to 20 µl bead bound DNA and 5 µl TS PCR oligo mix (0.5 µM, see Supplementary Table - Adapter sequences). PCR was performed as follows: 98°C for 45 seconds; 12-14 cycles of 98°C for 15 seconds, 60°C for 30 seconds, 72°C for 30 seconds; 72°C for 1 minute. DNA was isolated using 1.8 volumes of SPRIselect beads and eluted in 20 µl water. Concentration was determined using Qubit dsDNA BR Assay kit. The second capture was performed essentially like the first capture except a shorter hybridization time (24h instead of 72h) and fewer amplification cycles (6 instead of 12-14) were used. Eluted DNA was analyzed using Qubit dsDNA BR Assay kit and Bioanalyzer and used for sequencing.

Tissue-specific ChIP-seq on insulator proteins and H3K27ac

ChIP-seq was performed as described in Bonn et al.[18,19]. Embryos fixed in 1.8% formaldehyde (20 minutes) were dissociated using glass dounce homogenizers in ice-cold PBT (1xPBS + 0.1% Triton-X-100) with protease inhibitors (Complete Protease Inhibitors, Roche). The lysate was transferred into a reaction tube. After centrifugation at 400xg for 1 min at 4°C, the supernatant was transferred to a fresh tube and centrifuged again at 1,100xg for 10 minutes at 4°C. The pellet was homogenized again using a dounce homogenizer in cell lysis buffer (5 mM HEPES pH 8.0, 85 mM KCl, 0.5% IGEPAL CA-630 supplemented with Complete Protease Inhibitor, Roche), transferred to fresh tubes and centrifuged at 2,000xg for 4 minutes at 4°C. The nuclei were resuspended in 1 ml of ice-cold nuclear lysis buffer (50 mM HEPES pH 8.0, 10mM EDTA, 0.5% N-Laurylsarcosine supplemented with Complete Protease Inhibitor, Roche), and incubated on ice for 20 minutes. Nuclei were sonicated using a Bioruptor Pico (Diagenode) to generate 250-500 bp chromatin fragments (Settings: 12 cycles of 30 sec ON and 30 sec OFF). The chromatin was centrifuged at 16,000xg and the supernatant containing the chromatin was aliquoted into fresh tubes and stored at -80°C until use. The quality of the sheared chromatin was determined by agarose gel electrophoresis to observe chromatin fragment size distribution.

The antibodies used for ChIP-seq are listed in the Supplementary Table. The following antibodies, rabbit anti-CTCF, goat anti-Su(Hw) and rabbit anti-H3K27ac, were incubated overnight with chromatin in RIPA buffer (140mM NaCl, 10 mM Tris-HCl pH 8.0, 1 mM EDTA, 1% Triton-X-100, 0.1% SDS, 0.1% sodium deoxycholate, supplemented with Complete Protease Inhibitor, Roche) in a total volume of 900 ml. We used 2.5 µg chromatin for incubations with 1:900 dilution anti-CTCF antibody, 2 µg chromatin with 1:300 anti-Su(Hw) antibody, 1 µg chromatin with 1:900 anti-BEAF antibody, and 2 µg chromatin with 1:900 H3K27ac. The next day 25 ml of magnetic protein A/G beads (Dynabeads, Invitrogen, 10002D and 10004D) were washed with 1 ml of RIPA buffer and added to the IPs for an additional 3 hour incubation on a rotating wheel at 4°C. For the BEAF-32 ChIP, 25 µl of protein G beads were combined with 100 µl of the BEAF-32 antibody (DSHB, #1553420) and 300 ml RIPA buffer for 2 hrs. This was followed by two washes with RIPA and resuspension in 100 ml of RIPA buffer, which was added to the purified chromatin and incubated on a rotating wheel at 4°C overnight. The ChIPs were washed for 10 min on a rotating wheel: once with 1 ml RIPA buffer, 4 times with 1 ml RIPA-500 (500 mM NaCl, 10 mM Tris-HCl pH 8.0, 1 mM EDTA, 1% Triton X-100, 0.1% SDS, 0.1% Na-deoxycholate, supplemented with Complete Protease Inhibitor, Roche), once with 1 ml LiCl buffer (250 mM LiCl, 10 mM Tris-HCl pH 8.0, 1 mM EDTA, 0.5% IGEPAL CA-630, 0.5 % sodium deoxycholate) and twice with 1 ml TE buffer (10 mM Tris pH 8.0, 1 mM EDTA) on a magnetic rack in the cold room. The chromatin was then RNase-treated (final

concentration: 50 µg/ml in 100 µl volume; Cat. No. #10109142001, Roche), reverse cross-linked overnight with 0.5 mg/ml Proteinase K and 0.5% SDS at 65°C. The next day the DNA was purified using Phenol-Chloroform isolation and precipitated with ethanol, sodium acetate pH 5.3 and glycogen to obtain pure DNA. Library preparation was performed using the NEBNext® Ultra™ II DNA Library Prep Kit for Illumina® (NEB #E7645S), following the manufacturer's instructions. The quality of the libraries was assessed on a Bioanalyzer (Agilent), and libraries displayed a peak around 350-600 bp. For each ChIP at least two completely independent biological replicates were performed. ChIP-seq libraries were paired-end sequenced with 75 bp paired-end reads using Illumina NextSeq 500 platform at the EMBL Genomics Core Facility

DNA and RNA fluorescence in situ hybridization (FISH) in *Drosophila* embryos

All DNA, RNA and IF-DNA FISH experiments were performed on formaldehyde fixed (4% for 20 minutes at room temperature) embryos described in[2]. Probes are listed (Supplementary Table). Images were taken with a Leica SP8 confocal microscope using a 100x objective (HC PL APO CS2 100x / NA 1.4 / Oil), 405 nm laser, white-light laser (470-670 nm), and HyD detectors and Leica Application Suite X version 3.5.7.23225 (and previous). Z-stack step size was 100 or 200 nm. Multicolor DNA FISH signal distances were measured as described in[2] and taken from ventral nerve cord or brain regions (Elav+) as well as adjacent non-neuronal (Elav-) tissues from at least 3 different embryos for each stage. Images were analyzed using Fiji/ImageJ version 2.1.0./1.53c. DNA FISH distance measurements above 2 µm pair-wise distance were excluded from data analysis. For data visualization, violin/box plots were cut at 1 µm. Statistical analysis was performed with all data points (≤ 2 µm, equivalent to n indicated in the respective plots). Data were visualized and distance distributions compared using a two-sided Kolmogorov-Smirnov test on the combined data for each condition using R Studio (version RStudio 2022.07.1+554 and previous).

Whole-mount RNA *in-situ* hybridization experiments were carried out as described previously[20], with probes listed in the Supplementary Table (EST for in situ).

References

1. Cardozo Gizzi, A.M. *et al.* Microscopy-Based Chromosome Conformation Capture Enables Simultaneous Visualization of Genome Organization and Transcription in Intact Organisms. *Mol Cell* **74**, 212-222 e5 (2019).

2. Cavalheiro, G.R. *et al.* CTCF, BEAF-32, and CP190 are not required for the establishment of TADs in early Drosophila embryos but have locus-specific roles. *Sci Adv* **9**, eade1085 (2023).

3. Hug, C.B., Grimaldi, A.G., Kruse, K. & Vaquerizas, J.M. Chromatin Architecture Emerges during Zygotic Genome Activation Independent of Transcription. *Cell* **169**, 216-228 e19 (2017).

4. Sexton, T. *et al.* Three-dimensional folding and functional organization principles of the Drosophila genome. *Cell* **148**, 458-72 (2012).

5. Ghavi-Helm, Y. *et al.* Highly rearranged chromosomes reveal uncoupling between genome topology and gene expression. *Nat Genet* **51**, 1272-1282 (2019).

6. Mohana, G. *et al.* Chromosome-level organization of the regulatory genome in the Drosophila nervous system. *Cell* **186**, 3826-3844 e26 (2023).

7. Pollex, T. *et al.* Chromatin gene-gene loops support the cross-regulation of genes with related function. *Mol Cell* (2023).

8. Reddington, J.P. *et al.* Lineage-Resolved Enhancer and Promoter Usage during a Time Course of Embryogenesis. *Dev Cell* **55**, 648-664 e9 (2020).

9. Ghavi-Helm, Y. *et al.* Enhancer loops appear stable during development and are associated with paused polymerase. *Nature* **512**, 96-100 (2014).

10. Ramirez, F. *et al.* High-resolution TADs reveal DNA sequences underlying genome organization in flies. *Nat Commun* **9**, 189 (2018).

11. Van Bortle, K. *et al.* Insulator function and topological domain border strength scale with architectural protein occupancy. *Genome Biol* **15**, R82 (2014).

12. Jiang, N., Emberly, E., Cuvier, O. & Hart, C.M. Genome-wide mapping of boundary element-associated factor (BEAF) binding sites in Drosophila melanogaster links BEAF to transcription. *Mol Cell Biol* **29**, 3556-68 (2009).

13. Soshnev, A.A., Baxley, R.M., Manak, J.R., Tan, K. & Geyer, P.K. The insulator protein Suppressor of Hairy-wing is an essential transcriptional repressor in the Drosophila ovary. *Development* **140**, 3613-23 (2013).

14. Gambetta, M.C. & Furlong, E.E.M. The Insulator Protein CTCF Is Required for Correct Hox Gene Expression, but Not for Embryonic Development in Drosophila. *Genetics* **210**, 129-136 (2018).

15. Kaushal, A. *et al.* CTCF loss has limited effects on global genome architecture in Drosophila despite critical regulatory functions. *Nat Commun* **12**, 1011 (2021).

16. Roy, S., Jiang, N. & Hart, C.M. Lack of the Drosophila BEAF insulator proteins alters regulation of genes in the Antennapedia complex. *Mol Genet Genomics* **285**, 113-23 (2011).

17. Geyer, P.K. & Corces, V.G. DNA position-specific repression of transcription by a Drosophila zinc finger protein. *Genes Dev* **6**, 1865-73 (1992).

18. Bonn, S. *et al.* Tissue-specific analysis of chromatin state identifies temporal signatures of enhancer activity during embryonic development. *Nat Genet* **44**, 148-56 (2012).

19. Bonn, S. *et al.* Cell type-specific chromatin immunoprecipitation from multicellular complex samples using BiTS-ChIP. *Nat Protoc* **7**, 978-94 (2012).

20. Schor, I.E. *et al.* Non-coding RNA Expression, Function, and Variation during Drosophila Embryogenesis. *Curr Biol* **28**, 3547-3561 e9 (2018).
