# Supplementary material for: Enhancer–promoter interactions become more instructive in the transition from cell-fate specification to tissue differentiation
Source: Nat Genet. 2024 Mar 11;56(4):686–96. doi: 10.1038/s41588-024-01678-x (PMC11018526; doi:10.1038/s41588-024-01678-x)
Supplement: Supplementary file 2 — Reporting Summary [file 41588_2024_1678_MOESM2_ESM.pdf]

nature portfolio

Corresponding author(s): FURLONG

Last updated by author(s): Jan 18, 2024

Reporting Summary

Nature Portfolio wishes to improve the reproducibility of the work that we publish. This form provides structure for consistency and transparency in reporting. For further information on Nature Portfolio policies, see our Editorial Policies and the Editorial Policy Checklist.

Statistics

For all statistical analyses, confirm that the following items are present in the figure legend, table legend, main text, or Methods section.

n/a	Confirmed	
☐	☒	The exact sample size (*n*) for each experimental group/condition, given as a discrete number and unit of measurement
☒	☐	A statement on whether measurements were taken from distinct samples or whether the same sample was measured repeatedly
☐	☒	The statistical test(s) used AND whether they are one- or two-sided<br>*Only common tests should be described solely by name; describe more complex techniques in the Methods section.*
☐	☒	A description of all covariates tested
☐	☒	A description of any assumptions or corrections, such as tests of normality and adjustment for multiple comparisons
☐	☒	A full description of the statistical parameters including central tendency (e.g. means) or other basic estimates (e.g. regression coefficient) AND variation (e.g. standard deviation) or associated estimates of uncertainty (e.g. confidence intervals)
☐	☒	For null hypothesis testing, the test statistic (e.g. *F*, *t*, *r*) with confidence intervals, effect sizes, degrees of freedom and *P* value noted<br>*Give P values as exact values whenever suitable.*
☒	☐	For Bayesian analysis, information on the choice of priors and Markov chain Monte Carlo settings
☒	☐	For hierarchical and complex designs, identification of the appropriate level for tests and full reporting of outcomes
☐	☒	Estimates of effect sizes (e.g. Cohen's *d*, Pearson's *r*), indicating how they were calculated

Our web collection on statistics for biologists contains articles on many of the points above.

Software and code

Policy information about availability of computer code

Data collection	Sequencing: Illumina built-in software for HiSeq 2000, 4000 and NextSeq 500 sequencers (HCS v2.2.68, HCS v3.4.0 & NSS v2.2.0)<br>Imaging: Leica built-in software for Leica SP8 microscope (Leica Application Suite X 3.5.7.23225 and previous) and Zeiss built-in software for LSM 880 AIRY fast (ZEN 2.3SP1 and previous), Deconvolution: Huygens
Data analysis	The following version of software tools were used:<br>Image analysis: Fiji/ImageJ 2.1.0/1.53c, Java 1.8.0 [64bit] (and previous versions prior to updates)<br>RStudio2022.07.1 (and previous versions prior to updates)/R 4.2.1<br>Alignment of CaptureC data - Bowtie2=2.3.5<br>Identification of CaptureC pairs - hicup=0.6.1<br>Normalisation of interaction frequency - mgcv=1.8.22<br>Identification of signficant interactions - CHiCAGO=1.14.0<br>Identification of differential interactions - DESeq2=1.16.1<br>Alignment of ChIP data - Bowtie2=Galaxy Wrapper Version 2.3.4.2<br>Identification of ChIP peaks - Macs2=Galaxy Version 2.1.1.20160309.5

For manuscripts utilizing custom algorithms or software that are central to the research but not yet described in published literature, software must be made available to editors and reviewers. We strongly encourage code deposition in a community repository (e.g. GitHub). See the Nature Portfolio guidelines for submitting code & software for further information.

Data

Policy information about availability of data

All manuscripts must include a data availability statement. This statement should provide the following information, where applicable:

- Accession codes, unique identifiers, or web links for publicly available datasets
- A description of any restrictions on data availability
- For clinical datasets or third party data, please ensure that the statement adheres to our policy

All raw data (de-multiplexed files) for Capture-C and ChIP-seq were submitted to EMBL-EBI ArrayExpress (https://www.ebi.ac.uk/arrayexpress/browse.html) under accession numbers: E-MTAB-9310 (Capture-C) and E-MTAB-12639 (ChIP-seq). All processed data is available in Extended Data files 1-6, and will be available on the Furlong lab web page (http://furlonglab.embl.de/data), as standard practice in the group.
We also provide a user friendly searchable shiny app, which has all Capture-C interaction maps, and tissue specific insulator and H3K27ac ChIP-seq peaks, where one can visualise the data for all ~600 E/P baits: http://furlonglab.embl.de/data/E-P_CaptureC

Human research participants

Policy information about studies involving human research participants and Sex and Gender in Research.

Reporting on sex and gender	n.a.
Population characteristics	n.a.
Recruitment	n.a.
Ethics oversight	n.a.

Note that full information on the approval of the study protocol must also be provided in the manuscript.

Field-specific reporting

Please select the one below that is the best fit for your research. If you are not sure, read the appropriate sections before making your selection.

☒ Life sciences ☐ Behavioural & social sciences ☐ Ecological, evolutionary & environmental sciences

For a reference copy of the document with all sections, see nature.com/documents/nr-reporting-summary-flat.pdf

Life sciences study design

All studies must disclose on these points even when the disclosure is negative.

Sample size	No sample size calculation was performed. As commonly used, we performed two independent biological replicates (n=2 samples) per stage and tissue, each with 100 million nuclei for Capture-C to ensure enough biological complexity. 26 baits were added to both Capture libraries to assess reproducibility.
Data exclusions	For the Capture-C data, all data was deposited to ArrayExpress. For the analyses in the study, we excluded non-Mef2/non-Elav (Non-meso/non-neuro) Capture-C data for the majority of the analyses as indicated in the manuscript, as this represents very heterogenous cell types. But we included it in the data deposition in case it would be useful to others.<br>Three baits were excluded from the analyses as they failed the Capture C quality control.<br>For the ChIP-seq data we excluded 3 replicates (Neuro 6-8h Rep1 Su(Hw); Neuro 6-8h Rep1 CTCF; Neuro 10-12h Rep1 Su(Hw)) due to low read count and poor enrichment. Pseudo-replicates were used from the remaining replicates.
Replication	Capture-C: Each Capture-C replicate was generated from 100 milion sorted nuclei isolated from embryos fixed during different embryo collections. Thus, at least 2 independent biological replicates of each 100 mio sorted nuclei were generated for each stage and tissue (e.g. Replicate 1, 6-8h Mef2 and Replicate 2 6-8h Mef2).<br>ChIP-seq: As for Capture-C, chromatin for ChIP-seq experiments was generated from sorted nuclei isolated from embryos fixed during different embryo collections (biological replicates). At least two biological replicates were performed for each stage and tissue. Excluded replicates are mentioned above based on the QC of the ChIP-seq data.<br>IF-DNA-FISH: Distance measurements were performed with microscopy image stacks on hundreds of nuclei from at least 3 different embryos per stage from fixed over night collections. Comparisons of distances in different tissues (Elav positive versus Elav negative) were performed in adjacent tissues in the same embryos.
Randomization	Does not apply. No randomization was performed.
Blinding	Does not apply. Blinding was not performed.

Reporting for specific materials, systems and methods

We require information from authors about some types of materials, experimental systems and methods used in many studies. Here, indicate whether each material, system or method listed is relevant to your study. If you are not sure if a list item applies to your research, read the appropriate section before selecting a response.

Materials & experimental systems

n/a	Involved in the study
☐	☒ Antibodies
☒	☐ Eukaryotic cell lines
☒	☐ Palaeontology and archaeology
☐	☒ Animals and other organisms
☒	☐ Clinical data
☒	☐ Dual use research of concern

Methods

n/a	Involved in the study
☐	☒ ChIP-seq
☒	☐ Flow cytometry
☒	☐ MRI-based neuroimaging

Antibodies

Antibodies used	Primary antibodies: monoclonal mouse anti-Elav (DSHB Cat# Elav-9F8A9, RRID:AB_528217), rabbit anti-Mef2 (Furlong lab, EMBL Heidelberg, non-commercial/in-house), rabbit anti-Histone H3 (acetyl K27) (Abcam Cat# ab4729, RRID:AB_2118291), rabbit anti-CTCF (Rainer Renkawitz, non-commercial CTCF modENCODE antibody 2, RRID:AB_2616317), mouse anti-BEAF-32 (DSHB Cat# anti-BEAF, RRID:AB_1553420), goat anti-Su(Hw) (Pamela Geyer - University of Iowa, non-commercial su(Hw) modENCODE antibody 1, RRID:AB_2616308)<br>Secondary antibodies (Imaging/FANS): donkey anti-mouse Alexa Fluor 488 (Molecular Probes Cat# A-21202, RRID:AB_141607), goat anti-rabbit Alexa Fluor 555 (Thermo Fisher Scientific Cat# A-21428, RRID:AB_2535849), goat anti-mouse Alexa Fluor 647 (Thermo Fisher Scientific Cat# A-21240, RRID:AB_2535809)<br>Antibodies (in situ): anti-Digoxigenin-Peroxidase (Roche Cat# 11633716001, RRID:AB_514499), anti-Biotin-Peroxidase (Sigma-Aldrich Cat# A4541, RRID:AB_258185), anti-Fluorescein-Peroxidase (Roche Cat# AB840257, RRID:AB_2314405)
Validation	mouse anti-Elav (DSHB Elav-9F8A9) - https://dshb.biology.uiowa.edu/Elav-9F8A9; https://www.citeab.com/antibodies/150846-elav-9f8a9-elav-drosophila-protein<br>rabbit anti-Mef2 - e.g. doi:10.1016/j.devcel.2020.10.009 & doi:10.1016/j.devcel.2022.01.016<br>rabbit anti-Histone H3 (acetyl K27) - https://www.abcam.com/histone-h3-acetyl-k27-antibody-chip-grade-ab4729.html<br>mouse anti-BEAF-32 (DSHB anti-BEAF, #1553420) - https://dshb.biology.uiowa.edu/anti-BEAF; https://www.citeab.com/antibodies/150456-anti-beaf-beaf<br>rabbit anti-CTCF - https://www.encodeproject.org/antibodies/ENCAB863JZH/ & e.g. doi:10.1126/sciadv.ade1085<br>goat anti-Su(Hw) - https://www.encodeproject.org/antibodies/ENCAB959SMR/

Animals and other research organisms

Policy information about studies involving animals; ARRIVE guidelines recommended for reporting animal research, and Sex and Gender in Research

Laboratory animals	Species: Drosophila melanogaster; Strain: OregonR, w1118, yw and vas-Cas9 thereof derived mutants; age: Capture-C and ChIP-seq (2-3h, 6-8h, 10-12h after egg laying), IF-DNA-FISH and RNA in situ hybridization (over night collections of embryos); Sex: mixed male and female (total population)
Wild animals	Does not apply. This study does not involve animals collected in the wild.
Reporting on sex	Does not apply. Mixed populations of male and female embryos were used throughout the study.
Field-collected samples	Does not apply. This study does not contain materials collected in the field.
Ethics oversight	No ethical approval or guidance was required. Drosophila melanogaster is an invertebrate, and as such is not considered as an animal requiring ethics approval or guidance.

Note that full information on the approval of the study protocol must also be provided in the manuscript.

ChIP-seq

Data deposition

☐ Confirm that both raw and final processed data have been deposited in a public database such as GEO.

☒ Confirm that you have deposited or provided access to graph files (e.g. BED files) for the called peaks.

Data access links	https://www.ebi.ac.uk/biostudies/arrayexpress/studies/E-MTAB-9310?key=9abe1e3e-f26e-4a6d-84cb-0ef5b3fa555d

Data access links
May remain private before publication.

https://www.ebi.ac.uk/biostudies/arrayexpress/studies/E-MTAB-12639?key=9fad869f-e656-475c-aa76-0dfdf06be384

Files in database submission

All raw data (de-multiplexed files) for Capture-C and ChIP-seq were submitted to EMBL-EBI ArrayExpress (https://www.ebi.ac.uk/arrayexpress/browse.html) under accession numbers: E-MTAB-9310 (Capture-C data) and E-MTAB-12639 (ChIP-seq data).

Genome browser session
(e.g. UCSC)

We have generated a custom genome browser (using JBrowse) that shows all Capture C data in different tissues and time points, together with the CHIACGO tracks showing interactions above threshold, for all ~600 E/P bait.
Tracks showing the matching ChIP-seq data for insulator proteins are also be provided for visualization.
http://furlonglab.embl.de/data/E-P_CaptureC

Methodology

Replicates

All ChIP samples had two experimental replicates except su(Hw) IP in neuronal 6-8h and 10-12h tissue and CTCF IP in neuronal 6-8h tissue. In these three conditions one replicate was rejected during the QC analyses due to low read number and/or poor enrichment. The remaining replicate was randomly split into two pseudo replicates for subsequent processing.

Sequencing depth

Sample airing Length Reads Unique mapped pairs
WE_2-3h_Input_rep1 paired-end 42 31442314 -68,06%
WE_2-3h_BEAF_rep1 paired-end 40 19111809 -78,68%
WE_2-3h_BEAF_rep2 paired-end 40 25028071 -74,78%
WE_2-3h_CTCF_rep1 paired-end 40 24392740 -69,90%
WE_2-3h_CTCF_rep2 paired-end 40 23643432 -64,55%
WE_2-3h_K27ac_rep1 paired-end 42 44814514 -77,77%
WE_2-3h_K27ac_rep2 paired-end 42 58485830 -77,03%
WE_2-3h_SuHw_rep1 paired-end 40 20152346 -69,83%
WE_2-3h_SuHw_rep2 paired-end 40 24243833 -73,33%
elav_6-8h_Input_rep1 paired-end 42 22704250 -74,85%
elav_6-8h_Beaf_rep1 paired-end 40 16122330 -81,10%
elav_6-8h_Beaf_rep2 paired-end 40 24575959 -68,96%
elav_6-8h_CTCF_rep2 paired-end 40 16907731 -73,32%
elav_6-8h_K27ac_rep1 paired-end 42 35582051 -75,69%
elav_6-8h_K27ac_rep2 paired-end 42 39376533 -76,97%
elav_6-8h_SuHw_rep2 paired-end 40 15522691 -73,43%
elav_10-12h_Input_rep1 paired-end 42 26830345 -74,90%
elav_10-12h_BEAF_rep1 paired-end 40 18662520 -80,34%
elav_10-12h_BEAF_rep2 paired-end 40 25202788 -77,49%
elav_10-12h_CTCF_rep1 paired-end 40 18008953 -75,44%
elav_10-12h_CTCF_rep2 paired-end 40 25233621 -73,97%
elav_10-12h_K27ac_rep1 paired-end 42 38500591 -77,74%
elav_10-12h_K27ac_rep2 paired-end 42 45728156 -75,81%
elav_10-12h_SuHw_rep2 paired-end 40 22825253 -63,52%
Mef2_6-8h_Input_rep1 paired-end 42 24164448 -75,03%
Mef2_6-8h_Beaf_rep1 paired-end 40 15595939 -81,76%
Mef2_6-8h_Beaf_rep2 paired-end 40 20453079 -74,05%
Mef2_6-8h_CTCF_rep1 paired-end 40 14681566 -73,59%
Mef2_6-8h_CTCF_rep2 paired-end 40 18423776 -73,58%
Mef2_6-8h_K27ac_rep1 paired-end 42 45112970 -78,42%
Mef2_6-8h_K27ac_rep2 paired-end 42 46269589 -80,04%
Mef2_6-8h_SuHw_rep1 paired-end 40 14953099 -74,23%
Mef2_6-8h_SuHw_rep2 paired-end 40 16801271 -71,46%
Mef2_10-12h_Input_rep1 paired-end 42 24544345 -76,23%
Mef2_10-12h_BEAF_rep1 paired-end 40 18357032 -79,44%
Mef2_10-12h_BEAF_rep2 paired-end 40 23892310 -74,12%
Mef2_10-12h_CTCF_rep1 paired-end 40 26353619 -78,73%
Mef2_10-12h_CTCF_rep2 paired-end 40 16489278 -67,30%
Mef2_10-12h_K27ac_rep1 paired-end 42 31186278 -77,97%
Mef2_10-12h_K27ac_rep2 paired-end 42 39184106 -73,87%
Mef2_10-12h_SuHw_rep1 paired-end 40 19850949 -72,76%
Mef2_10-12h_SuHw_rep2 paired-end 40 18139793 -69,66%

Antibodies

rabbit anti-Histone H3 (acetyl K27) (Abcam, ab4729), rabbit anti-CTCF (Renkawitz lab, University of Giessen), mouse anti-BEAF-32 (DSHB anti-BEAF, #1553420), goat anti-Su(Hw) (Geyer lab, University of Iowa)

Peak calling parameters

ChIP peaks were identified using Mac2 software using the call-summits flag and an FDR threshold of 0.05. All summits of the insulator proteins (BEAF-32, CTCF and su(Hw)) were grouped so that each summit within a group was within 50bp of its nearest neighbor and all summits within a group was 50bp, or further, from the summits of nearest neighboring group. These grouped summits are the initial insulator peaks. The initial insulator peaks were filtered to remove those that do not contain a summit for both replicates of at least one insulator protein. The final insulator peak are derived by expanding those filtered peaks that are less than 300bp in width to a maximum size of 300bp whilst ensuring no expansion caused an overlap with the nearest neighbor. Individual insulator proteins were said to bind the insulator peak if there was a summit for both replicates of that insulator protein within the peak. H3K27ac peaks were derived in an identical manner to the insulator peaks but utilizing just the two replicates of the H3K27ac ChIP. All the final H3K27ac peaks contain at least one summit from each of the two replicates.

Data quality	After initial QC based on read number and mappability, peaks were called using an FDR of 0.05. Insulator peaks were filtered to remove those that do not contain a summit for both replicates of at least one insulator protein. Individual insulator proteins, or H3K27ac, were said to bind (or be present) if there was a summit for both replicates of that insulator protein (or H3K27ac) within the peak.
Software	Alignment of ChIP data - Bowtie2=Galaxy Wrapper Version 2.3.4.2<br>Identification of ChIP peaks - Macs2=Galaxy Version 2.1.1.20160309.5
